# Meta-Analysis of Reciprocal Linkages between Temperate Seagrasses and Waterfowl with Implications for Conservation

**DOI:** 10.3389/fpls.2017.02119

**Published:** 2017-12-22

**Authors:** Nicole M. Kollars, Amy K. Henry, Matthew A. Whalen, Katharyn E. Boyer, Mathieu Cusson, Johan S. Eklöf, Clara M. Hereu, Pablo Jorgensen, Stephanie L. Kiriakopolos, Pamela L. Reynolds, Fiona Tomas, Mo S. Turner, Jennifer L. Ruesink

**Affiliations:** ^1^Center for Population Biology, University of California, Davis, Davis, CA, United States; ^2^Committee on Evolutionary Biology, The University of Chicago, Chicago, IL, United States; ^3^Department of Evolution and Ecology, University of California, Davis, Davis, CA, United States; ^4^Hakai Institute, Vancouver, BC, Canada; ^5^Romberg Tiburon Center and Department of Biology, San Francisco State University, Tiburon, CA, United States; ^6^Département des Sciences Fondamentales & Québec-Océan, Université du Québec à Chicoutimi, Chicoutimi, QC, Canada; ^7^Department of Ecology, Environment and Plant Sciences, Stockholm University, Stockholm, Sweden; ^8^Facultad de Ciencias Marinas, Universidad Autónoma de Baja California, Ensenada, Mexico; ^9^Geomare AC, Ensenada, Mexico; ^10^Department of Fisheries and Wildlife, Oregon State University, Corvallis, OR, United States; ^11^Data Science Initiative, University of California, Davis, Davis, CA, United States; ^12^Instituto Mediterráneo de Estudios Avanzados, Universitat de les Illes Balears – Consejo Superior de Investigaciones Científicas, Esporles, Spain; ^13^Department of Biology, University of Washington, Seattle, WA, United States

**Keywords:** herbivory, geese, productivity, swans, top-down effects, *Zostera*

## Abstract

Multi-trophic conservation and management strategies may be necessary if reciprocal linkages between primary producers and their consumers are strong. While herbivory on aquatic plants is well-studied, direct top-down control of seagrass populations has received comparatively little attention, particularly in temperate regions. Herein, we used qualitative and meta-analytic approaches to assess the scope and consequences of avian (primarily waterfowl) herbivory on temperate seagrasses of the genus *Zostera*. Meta-analyses revealed widespread evidence of spatio-temporal correlations between *Zostera* and waterfowl abundances as well as strong top-down effects of grazing on *Zostera*. We also documented the identity and diversity of avian species reported to consume *Zostera* and qualitatively assessed their potential to exert top-down control. Our results demonstrate that *Zostera* and their avian herbivores are ecologically linked and we suggest that bird herbivory may influence the spatial structure, composition, and functioning of the seagrass ecosystem. Therefore, the consequences of avian herbivory should be considered in the management of seagrass populations. Of particular concern are instances of seagrass overgrazing by waterfowl which result in long-term reductions in seagrass biomass or coverage, with subsequent impacts on local populations of waterfowl and other seagrass-affiliated species. While our results showed that bird density and type may affect the magnitude of the top-down effects of avian herbivory, empirical research on the strength, context-dependency, and indirect effects of waterfowl–*Zostera* interactions remains limited. For example, increased efforts that explicitly measure the effects of different functional groups of birds on seagrass abundance and/or document how climate change-driven shifts in waterfowl migratory patterns impact seagrass phenology and population structure will advance research programs for both ecologists and managers concerned with the joint conservation of both seagrasses and their avian herbivores.

## Introduction

Many management policies consider the conservation of one group of species independent of their interactions with other species of conservation concern. However, populations are frequently linked in natural systems (e.g., predators and prey; Stenseth et al., 1997) and understanding how changes in one taxon affect another remains a key challenge in conservation and ecology. We may expect interactions between taxa to be strong in aquatic systems where plants and seaweeds act as foundation species (*sensu* Dayton, 1973) for a diversity of organisms that utilize macrophytes for habitat and/or as a primary food source (Bakker et al., 2016). Within these systems, strong linkages between macrophytes and their consumers can arise when consumers depend on the macrophytes, but also exert strong top-down control on macrophyte populations through extensive tissue damage and biomass removal (reviewed in Poore et al., 2012; Wood et al., 2012, 2017; Bakker et al., 2016). In cases where both the primary producer and consumer are a focus of conservation, recognition of these reciprocal linkages can inform the effective management of both taxa.

In temperate systems, scientists and managers use coastal waterfowl to motivate the protection of seagrass beds, given an understanding that seagrass provides high-value resources which supports migratory and breeding activities (Sedinger et al., 2011; Schamber et al., 2012). Climate change, over-hunting, and habitat destruction all threaten waterfowl populations (e.g., Ward et al., 2005). Declines in *Zostera* spp. (hereafter *Zostera*) can reduce waterfowl carrying capacities or necessitate a behavioral shift to other sites or resources. In the 1930s, an epidemic of seagrass wasting disease (*Labyrinthula zosterae*) removed the majority of *Zostera marina* from North Atlantic coastlines in America and Europe, followed by a collapse in brant (*Branta bernicla* sp.) populations (Addy and Aylward, 1944). Although brant were thought to be obligate consumers of *Z. marina* (reviewed by Muehlstein, 1989), the geese have since recovered with a shift in diet to agricultural lands (Moore et al., 2004; Inger et al., 2006a,b). The Pacific brant (*Branta bernicla nigricans*) largely remains an obligate *Z. marina* consumer during migration and over winter, but will occasionally consume other marine primary producers such as *Ulva* (Hereu and Jorgensen, personal observation) and feed in Arctic salt marshes while nesting in the summer (Ward et al., 2005). Local losses of *Zostera* may be compensated through behavioral flexibility, which enables brant to use other stopover or wintering locales (Sedinger et al., 2006) rather than necessitating dietary flexibility or resulting in population-level declines. Nevertheless, the local consequences of *Zostera* losses appear dramatic, as predicted by models relating brant carrying capacity to *Zostera* abundance (Stillman et al., 2015) and evidenced by the decline in brant geese populations in Morro Bay after *Z. marina* essentially disappeared in the mid-2000s (Harencar et al., personal communication).

**FIGURE 1:**
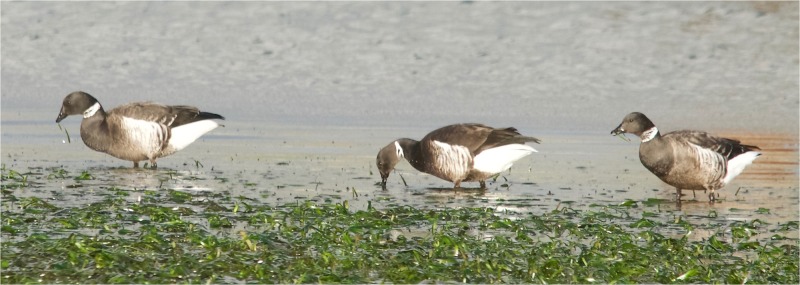
Pacific brant geese (*Branta bernicla nigricans*) feeding on *Zostera marina* in Bodega Bay, California. Photo credit: Gabriel Ng.

While conservation of avian herbivores includes consideration of their seagrass food, the linkage between seagrasses and their herbivores is typically not considered in the management of temperate seagrass populations (Maxwell et al., 2016). Several waterfowl species consume *Zostera* (**Figure 1**), but their top-down effects on seagrass bed dynamics is often assumed to be minor due to the birds’ temporally limited residence and rapid productivity in *Zostera* (Valentine and Heck, 1999; Ganter, 2000; Valentine and Duffy, 2007). Studies of top-down effects by grazing birds on seagrasses typically aim to estimate the impact of herbivory as reductions of standing stock biomass or production (Thayer et al., 1984; Bakker et al., 2016) and results show that annual *Zostera* productivity can be two or three times greater than peak standing stock biomass (McRoy, 1966, 1970; Penhale, 1977; Thom and Hallum, 1990; Kaldy, 2006). Furthermore, grazing pressure is neither uniform temporally nor spatially, and total impacts of waterfowl on annual production can be minimal (as low as 3%; Thayer et al., 1984). Thus, the perspective first proposed by Kikuchi and Peres (1977) that most vegetative material in temperate seagrass beds is consumed through detrital pathways remains widely held.

Despite this prevailing view of minor top-down impacts of waterfowl on temperate seagrasses, reductions in plant standing crop by waterfowl could be significant where waterfowl biomass per unit area is high, where alternative foraging areas are scarce, or when waterfowl mobility between sites is limited (Thayer et al., 1984; Wood et al., 2012, 2017). Several case studies highlight that birds have contributed to large-scale losses of seagrass beds. Many seagrass-consuming birds dig up and eat the rhizomes or shoot meristems, which can damage the bed and inhibit recovery (Thayer et al., 1984). In San Francisco Bay, California, migrating Canada geese (*Branta canadensis*) remove all the eelgrass shoots in a shallow bed upon their arrival in the fall. Experiments showed that unless the geese are excluded, the bed must recover by seedling recruitment each spring (Kiriakopolos, 2013). Reliance on seeds is risky; Canada geese grazing led to local extinction of eelgrass in a New England bed where seedling recruitment was minimal (Rivers and Short, 2007). In New Zealand, intense grazing by black swans (*Cygnus atratus*) can remove 19–20% of the annual *Zostera* production, resulting in a 43–69% decrease in the standing biomass during the following growing season (Dos Santos et al., 2012, 2013). These studies highlight the potential for waterfowl to exert strong top-down pressure on seagrass ecosystems, but the generality of such phenomena is unknown and there remains little systematic understanding of the conditions under which bird herbivory alters the structure, function, or dynamics of temperate eelgrass beds.

Here we address the assumption that linkages between vertebrate herbivores and temperate seagrasses are weak by reviewing the literature to explicitly quantify the strength of waterfowl interactions with seagrass of the genus *Zostera* at temperate latitudes (30–60° north and south of the equator). Specifically, we examine the prevalence, scope, impact, and consequences of waterfowl herbivory on *Zostera* by addressing the following research questions:

1) What is the relationship between *Zostera* abundance and waterfowl abundance?
2) How strong are the consumptive effects of waterfowl on *Zostera* abundance?
3) How many avian species use *Zostera* as a resource?

We conclude our analysis with a broader view of system-wide consequences of waterfowl herbivory on seagrass ecosystems, which extend beyond reductions in seagrass abundance. Finally, we consider the implications of these reciprocal linkages for the conservation of both waterfowl and seagrass species as well as identify important avenues for future research in this area.

## Methods

We used a systematic literature review in combination with meta-analytic techniques to (1) quantitatively describe the correlation between *Zostera* abundance and waterfowl abundance, (2) measure the effect size of waterfowl herbivory on *Zostera* abundance, and (3) compile a list of avian species known to consume *Zostera*. On November 15, 2016 we searched ISI Web of Science using the terms “*Zostera* AND (bird^∗^ AND (herbiv^∗^ OR graz^∗^) OR (waterfowl OR goose OR geese OR brant).” To uncover additional relevant literature, we applied a forward and backward search from those citations, consulted earlier reviews (Valentine and Heck, 1999; Olsen, 2015; Wood et al., 2017), and contacted researchers with coastal waterfowl expertise (P. Clausen, Denmark; M. Hori, Japan; A. Olsen, United States). We also included an additional unpublished data set (C. M. Hereu and P. Jorgensen, experiment performed November 2011 to November 2012). For all research objectives, we excluded studies that did not assess natural grazing by waterfowl (e.g., researcher-simulated herbivory by clipping leaves or digging pits).

1) What is the relationship between *Zostera* abundance and waterfowl abundance?

The analysis of the shared variation in waterfowl and *Zostera* required at least four time points or locations. Studies that met this criterion ranged widely in the scope of observation; some spatial studies covered bird use of a single tidal flat with heterogeneous *Zostera* distribution (e.g., Percival and Evans, 1997; Dixon, 2009), whereas others compared tidal flats spanning hundreds or thousands of kilometers (e.g., Clausen and Percival, 1998; Moore et al., 2004). Most temporal data sets had annual time steps, in which birds and *Zostera* were recorded using similar methods over as much as three decades, but we also included one study with monthly time steps, where other evidence indicated that changes in bird abundance were not due to seasonal migration but rather to behavioral choices among a range of possible habitats (Tinkler et al., 2009). Variability in bird use due to tidal cycle changes in water level is well established (Clausen, 2000) but at too fine of a temporal scale for relevance in this meta-analysis. At large temporal or spatial scales, birds were typically assessed for bird-days (a metric of population-level habitat use at a site) integrated over a season or for peak numbers. When data sets were of smaller temporal or spatial scales, birds were typically tracked for behavior, such as foraging time or fecal dropping rate. Large-scale data sets for *Zostera* included estimates of area covered (e.g., at the scale of km^2^), whereas small-scale data sets for *Zostera* had sample units of percent cover or biomass per area (usually per m^2^). We calculated effect size using Pearson’s correlation coefficient (*r*) between waterfowl and *Zostera*, followed by transformation with Fisher’s *Z* ($Z=0.5*In\left(\frac{1+r}{1-r}\right)$) and a calculated standard deviation derived from sample size ($VZ=\frac{1}{n-3}$). We calculated an effect size for each waterfowl species separately if the study reported multiple species (Percival et al., 1998; Petersen et al., 2008; Balsby et al., 2017).

We applied a linear mixed effects model to the transformed correlation coefficients, with study considered a random effect to account for multiple bird species measured across the same *Zostera* samples. We assessed the statistical importance of two potential predictor variables: first, the amount of variation in the abundance of *Zostera* as calculated by the coefficient of variation among samples within a study (standard deviation divided by the mean; CV); and second, whether the study was carried out within a bay or year (defined as a small scale) or across bays or years (large scale). We tested how well the inclusion of the predictor variables explained the heterogeneity in the data using a Cochran’s *Q*-test, which tests the null hypothesis of homogeneity among samples. Analysis was carried out using R software (R Core Team, 2015) with the function rma.mv in the metafor package (Viechtbauer, 2010).

2) How strong are the consumptive effects of waterfowl on *Zostera* abundance?

To examine the top-down effects of waterfowl on *Zostera*, we only included studies that measured the response of *Zostera* abundance variables to the presence and absence of waterfowl (i.e., caging experiments, or observational experiments where waterfowl presence and absence varied spatially or temporally). Response variables included metrics that reflect the abundance of *Zostera* at the plot level (e.g., number of shoots in a 1-m^2^ area, canopy height, percent coverage of vegetation, and aboveground and belowground biomass). Within a study, we extracted data points across multiple response variables and multiple data points. We treated publications that reported results from multiple study sites as independent only if the seagrass beds were non-overlapping (Dixon, 2009). We excluded records if the variance could not be extracted with the information provided in the publication (Madsen, 1988; Nacken and Reise, 2000, and certain data points within Tubbs and Tubbs, 1983). A subset of data points within one study (*n* = 14 data points in Kiriakopolos, 2013) had a mean and standard deviation value equal to 0 in both the presence and absence of waterfowl, so these points were removed. Effect sizes were calculated as the standardized mean difference ($Hedges’d,d=\frac{M_{1}-M_{2}}{{SD}_{*pooled}},{SD}_{*pooled}=\sqrt{\frac{(n_{1}-1){SD}_{1}^{2}+(n_{2}-1){SD}_{2}^{2}}{n_{1}+n_{2}-2}},$ Hedges and Olkin, 1985; with confidence intervals corrected for heteroscedastic population variances between groups, Bonett, 2009) using the function escalc from the package metafor (Viechtbauer, 2010) in R (R Core Team, 2017). We only retained the maximum effect size from each study for further analysis. We excluded one outlier from Rivers and Short (2007) because including the maximum effect size in regression analyses (see below) yielded standardized residuals greater than two standard deviations away from the mean. Instead, we retained the second largest maximum effect size for this study.

We first used a random-effects model to estimate the overall effect size of waterfowl on *Zostera* abundance and test for heterogeneity in effect sizes among studies. Analyses used the rma function in R metafor package (Viechtbauer, 2010). To test for potential publication bias among our set of studies, we used a funnel plot to visualize effect size versus standard error and statistically tested for asymmetry using the function regtest in metafor. There was significant asymmetry among our studies (*z* = -5.99, *p*-value <0.001; **Supplementary Figure S1**). Though this asymmetry may result from chance alone given the low number of studies in the dataset, it is plausible that our set of studies does not represent an unbiased sample (Koricheva et al., 2013). The bias may arise from publication bias against non-significant results, system heterogeneity across studies, or that ecologists tend to set-up labor-intensive experiments only when they expect to see a result. With this caveat in mind, we pursued a simple analysis of top-down effects of waterfowl on eelgrass from the data currently reported in the literature.

We explored potential explanatory variables that could account for the observed heterogeneity among the effect sizes using linear mixed-effects models. Explanatory variables included latitude, longitude, waterfowl species, bird density (measured as individuals per hectare), *Zostera* response variable, *Zostera* species, area sampled, and the time elapsed between when the experiment began and when the maximum effect was observed. For studies that only reported experiment start dates and observations as a month in a year, we coded the date as the first day of the month and calculated the time elapsed since the experiment began in number of days. Several studies involved either a goose or swan combined with a duck species (e.g., Tubbs and Tubbs, 1983; Madsen, 1988; Gayet et al., 2012) but never a goose and swan together. No study included a duck species exclusively, so for waterfowl species, we further classified whether the study included a goose or a swan species. Similarly, we grouped the *Zostera* response variable into categories that classified whether the response estimated aboveground biomass, belowground biomass, or total biomass. We extracted data on explanatory variables directly from the study. We tested whether each predictor variable influenced the effect size in separate models by including the predictor term in the rma function described above. No predictors were combined in a single model due to limitations of sample size. We estimated the fit of each individual model to the data using Akaike information criteria corrected for small sample size (AICc) along with an accompanying pseudo *R*^2^ statistic. We calculated pseudo *R*^2^ as the explained proportion of heterogeneity in effect sizes from the mixed-effects model with the explanatory variable relative to the random-effects model with no explanatory variable (Raudenbush, 2009).

3) How many avian species use *Zostera* as a resource?

Bird diets were compiled from the previously described literature review, including backward and forward citation searches from diet data compiled in Olsen (2015). Data were included from studies on gut contents, fecal analyses, and observations of feeding, as well as consulting more general field guides (Poole, 2005). For birds consuming *Zostera*, their use was assessed in terms of what fraction of their diet contained *Zostera* at particular observation times. These were categorized as “dominant,” in which *Zostera* composed 50–100% of diet during at least one season; “frequent,” in which *Zostera* is 5–50% and unlikely to be a dominant food source; or “incidental” in which <5% or rare observations of consumption occur (**Table 3**). Where noted in studies, we also recorded the seasonal stage of primary consumption (i.e., migration, overwintering) and the part of the plant consumed (i.e., leaves, rhizomes). See **Supplementary Table S2** for details.

## Results

**FIGURE 2:**
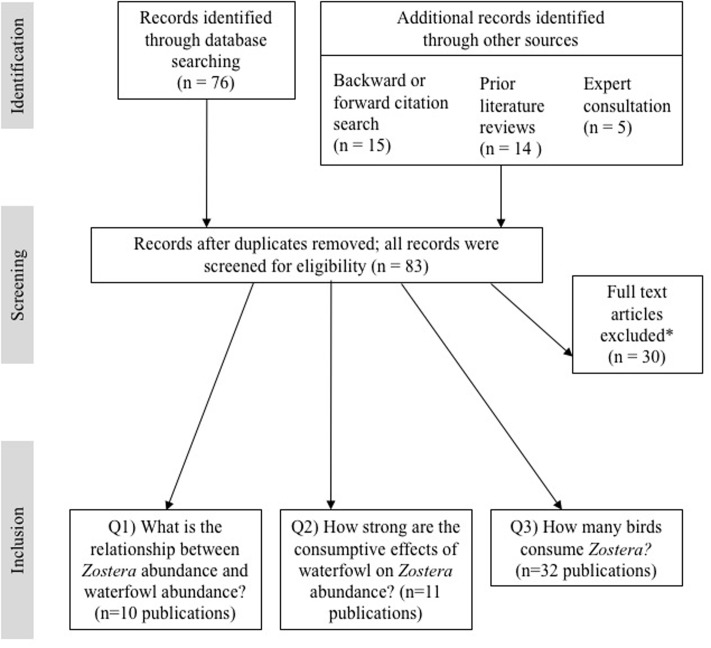
Flow-diagram of the screening process for the systematic literature review. ^∗^Publications were excluded if they did not meet one or more of the following criteria: (Q1) data series of both *Zostera* and bird abundance with at least four sample points; (Q2) comparison of *Zostera* abundance with and without birds (i.e., caging experiments or spatial/temporal comparisons); and (Q3) records of diet composition. See Sections “Methods” and “Results” for further details and **Supplementary Table S1** for a complete list of the screened publications.

**FIGURE 3:**
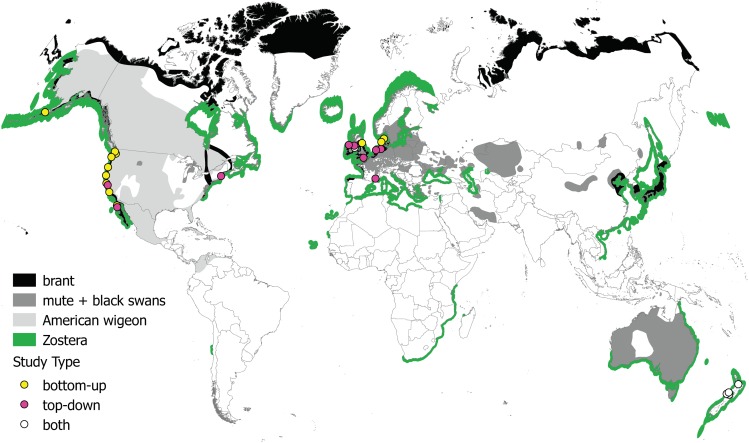
Map of study sites and organism distributions. Studies are categorized by the type of data used in our meta-analyses: abundance correlation (i.e., bottom-up), top-down, or both. The distribution of all species in the genus *Zostera* is shown in green, and distributions of birds whose diets consist of 50–100% *Zostera*, seasonally or year-round are shown in black (brant), dark gray (swans), and light gray (American wigeon). Distribution data were obtained from the International Union for Conservation of Nature (IUCN, 2017). Map made in QGIS (2017) using a circular projection (van der Grinten, 1904).

We identified 76 papers via Web of Science and an additional seven papers through forward/backward citation searches and expert consultation. This yielded a total candidate study list of 83 papers. From this list, we retained all publications that satisfied the unique criteria we developed for each analysis (see section “Methods”). This resulted in 10 publications used for investigating the relationship between waterfowl and *Zostera* abundance, 11 publications used for the top-down meta-analysis, and 32 publications used for the diet assessment (see **Figure 2** and **Supplementary Table S2**). Datasets used in the abundance relationship and the top-down analyses included four *Zostera* species: *Z. japonica* (one publication), *Z. marina* (10 publications, including two under the name *Z. angustifolia*), *Z. noltei* (nine publications), and *Z. muelleri* (two publications). Five publications included multiple species of *Zostera* (*Z. noltei* and *Z. marina*). **Figure 3** illustrates the distribution of the *Zostera* genus, the distribution of the primary waterfowl herbivores, and the study locations from the meta-analyses.

1) What is the relationship between *Zostera* abundance and waterfowl abundance?

**FIGURE 4:**
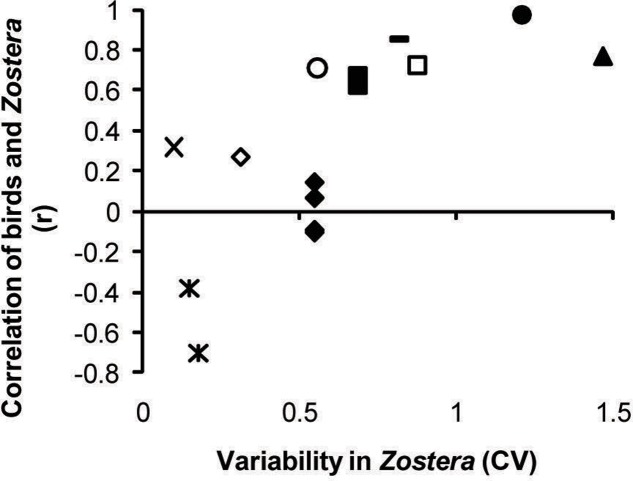
Pearson’s *r* showing the strength of correlation between birds and *Zostera* in observations across space or time. Positive correlations emerge where *Zostera* abundance is highly variable over space or time, with this spatiotemporal variability plotted on the *x*-axis as the coefficient of variation across *Zostera* samples. Small scale studies (within bay or within year): 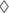, Dixon, 2009; 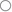, Percival and Evans, 1997; 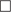, Tinkler et al., 2009. Large scale studies (across bays or years): ×, Wilson and Atkinson, 1995; ^∗^, Percival et al., 1998; 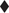, Balsby et al., 2017; 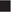, Petersen et al., 2008; 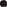, Dos Santos et al., 2012; 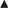, Moore et al., 2004. Raw correlations between bird and *Zostera* abundances for each study are provided in **Supplementary Figure S2**.

Waterfowl and *Zostera* abundance tended to track each other spatiotemporally (positive correlations, *r* > 0), but only when variability in *Zostera* was substantial. Accordingly, *Zostera* CV was a significant modifier of the waterfowl–*Zostera* correlation, and the amount of correlation between the two taxa increased with variability in *Zostera* (**Table 1** and **Figure 4**). The data sets from which correlation coefficients and *Zostera* CV were calculated are presented in the **Supplementary Figure S2**. When the coefficient of variation for *Zostera* was very low, we observed several negative correlations between waterfowl and *Zostera* abundance in one study (Percival et al., 1998), possibly reflecting depletion by birds. While *Zostera* variation significantly predicted the correlation, the scale of the study did not (**Table 1**). However, with the inclusion of these two predictors, Cochran’s *Q*-test showed no additional heterogeneity in the meta-analysis (*QE*_12_ = 14.45, *p*-value = 0.27).

**Table 1:** Model results for meta-analysis of Pearson’s correlations of birds and *Zostera*, transformed by Fisher’s *r*-to-*Z* transformation.

	Estimate	SE	*Z*-value	*p*-value	Lower confidence interval	Upper confidence interval
Intercept	-0.48	0.32	-1.49	0.14	-1.12	0.15
*Zostera* CV	1.32	0.43	3.06	0.002^∗∗^	0.47	2.17
Large or small scale	0.45	0.27	1.67	0.095	-0.079	0.99


*A multilevel meta-analysis was applied to account for studies in which several bird species were counted. *Zostera* CV represents the coefficient of variation in the abundance of *Zostera* across samples. Small scale studies occurred within bays or within years and generally examined behavioral responses of birds to *Zostera*, whereas large scale studies spanned bays or years and assessed birds by seasonal use-days or by peak counts.*

2) How strong are the consumptive effects of waterfowl on *Zostera* abundance?

**FIGURE 5:**
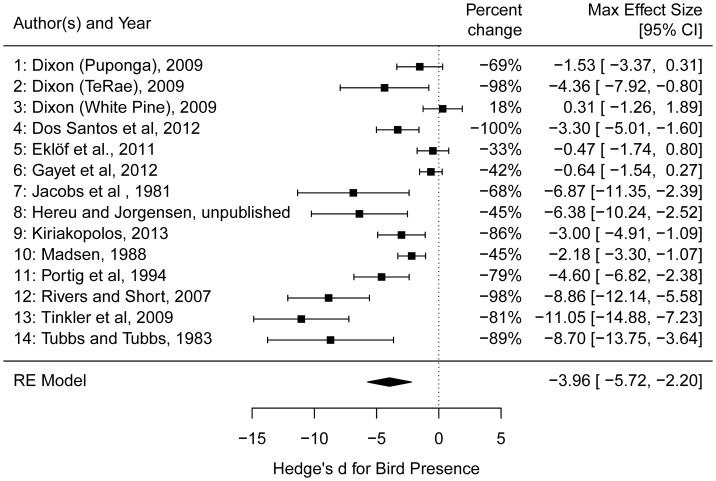
Forest plot of the maximum effect sizes (Hedges’ *d*) and 95% confidence intervals from the 14 independent measurements from 11 studies used in the top-down analysis. Percent changes of the mean biomass in the absence of birds from the presence of birds is also provided. Dixon (2009) performed experiments at three unique sites and we treated each site as an independent study (site name listed in parentheses). The RE model is the random effects model produced by the rma function in the package metafor and shows the estimate and confidence interval of the overall effect of waterfowl presence on *Zostera* abundance.

**FIGURE 6:**
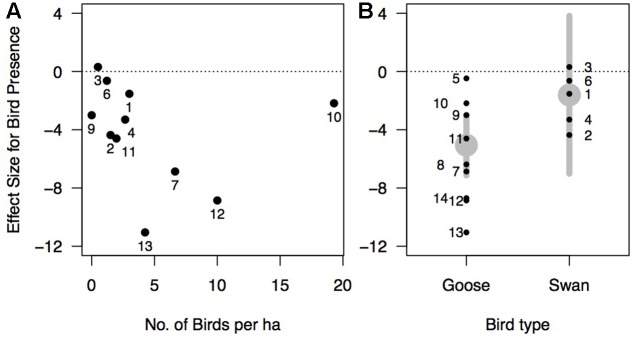
Effect size of waterfowl on eelgrass (Hedges’ *d*) as a function of: **(A)** bird density (number of birds per hectare). The model describing this relationship was the most supported in our model set based on Akaike information criterion (**Table 2**). Note: three studies were excluded because they did not report bird density (Tubbs and Tubbs, 1983; Eklöf et al., 2011; Hereu and Jorgensen unpublished data). **(B)** Bird type (goose or swan). This model is the second model most supported in our model set (**Table 2**). Gray points represent the effect size estimated by the model with gray lines showing the 95% confidence interval. In panels **(A,B)**, points are numbered by study as in **Figure 5**.

Effects of waterfowl on *Zostera* ranged from strongly negative (e.g., an 81% reduction in rhizome biomass from Tinkler et al., 2009) to slightly positive (an 18% biomass increase in rhizome biomass from Dixon, 2009 at one site) with an overall effect size estimated at -3.96 (95% confidence interval of -5.72 to -2.20) (**Figure 5**). The random effects model without predictors showed a large amount of heterogeneity in effect sizes among the studies (*Q*_13_ = 84.78, *p*-value <0.001), leading us to consider potential predictor variables to explain this heterogeneity (see section “Methods”). Among the models tested, the model of effect size as a function of bird density had the greatest AICc support, but explained only 3.5% of the variance among studies (**Table 2**). The magnitude of the negative effect of waterfowl on *Zostera* abundance tended to increase with increasing bird density (**Figure 6A**), though the slope was not statistically significant (estimate = -0.11, *Z* = -0.64, *p*-value = 0.53; note that excluding Madsen, 1988 results in a significant slope with an estimate = -0.95, *Z* = -3.57, *p*-value = 0.0004). Other models had very little support, but we report two models with significant predictors that ranked second and third in our model comparison according to the AICc values. Bird arrival and bird type (goose versus swan) both explained some of the heterogeneity in effect sizes (27.2 and 18.5%, respectively), but they largely contained the same information. All geese arrived in autumn with one exception (Kiriakopolos, 2013), and all swans arrived in summer, therefore we cannot distinguish these two models. Overall, studies with goose species had stronger negative effects on *Zostera* than studies involving swan species (**Figure 6B**).

**Table 2:** Akaike information criteria (AIC) results for a model set used to test for heterogeneity in effect sizes among studies investigating the consumptive effects of waterfowl on *Zostera* abundance.

Model	AICc	Delta AICc (Δ*_i_*)	Akaike weight (*w_i_*)	*R*^2^ (%)
Bird density	59.15	0.00	0.982	3.5
Bird arrival	69.36	10.21	0.006	31.3
Goose versus swan	69.93	10.78	0.004	30.3
Longitude	70.54	11.39	0.003	24.6
Latitude	71.97	12.82	0.002	15.1
Area sampled	72.85	13.70	0.001	4.4
Time since start of experiment	73.21	14.06	0.001	5.6
Above versus below	73.88	14.72	0.001	15.7
Intercept only	74.29	15.14	0.001	0.0
*Zostera* species	78.30	19.15	<0.001	27.4
Bird species	228.29	168.44	<0.001	81.5
*Zostera* response	233.08	173.92	<0.001	25.0


*The intercept only model was calculated using a random effects model on the mean effect size (yi; calculated as Hedges’ *d*) and its corresponding variance (xi). Due to low sample size, each modifier was tested in a model independent of other modifiers. AICc shows the AIC values corrected for small sample sizes. Delta AICc measures the goodness of fit for the model.*

3) How many avian species use *Zostera* as a resource?

The number of avian species documented as consumers of *Zostera* greatly exceeds the number that have been studied for top-down and bottom-up effects. In total, we identified 39 species and subspecies of waterfowl that included *Zostera* in their diet at any one location or time point: nine dabbling ducks, 16 diving ducks, six geese (including three subspecies of brant, differentiated by distinct migratory pathways and feeding behaviors), five swans, two rails, and one wader species (**Table 3** and **Supplementary Table S2**). Nearly all identified species are generalist herbivores that consume a variety of plant and non-plant food resources aside from *Zostera. Zostera* is a dominant component of the diets of six species (including the three subspecies of brant), frequent in the diets of 20 species, and rarely consumed by 13 additional waterfowl (**Table 3**). Except for the mute swan (*Cygnus olor*), all the heaviest consumers are represented in our correlation and top-down analyses. The majority of infrequent and rare consumers are dabbling and diving ducks. Comparing by species of seagrass consumed, *Z. marina* is consumed by the greatest number of bird species (27, many from Cottam, 1939), followed by five species for *Z. japonica*, three for *Z. noltei*, one for *Z. muelleri*, and ten in which only *Zostera* spp. was noted (**Table 3** and **Supplementary Table S2**). While we had anticipated that different taxa might use different feeding techniques (e.g., grazing of leaves versus grubbing for rhizomes), we found that different sources documented different feeding behaviors for the same species. These changes in feeding behavior might reflect variation in whether leaves or rhizomes have higher nutritional value, or when loss of aboveground biomass necessitates a shift to grubbing for rhizomes (Mathers et al., 1998). Of the 39 avian species consuming *Zostera*, 13 are noted to interact with the seagrass primarily during a specific season, either migration or overwintering (**Supplementary Table S2**).

**Table 3:** Diversity of avian herbivory on *Zostera*.

Avian taxa	Diet can be 50–100% *Zostera*, seasonally or year-round	*Zostera* frequent in diet (5–50%) but rarely dominant	Infrequent or incidental consumption
Dabbling ducks	*Anas americana*	*Anas acuta*, *Anas penelope* (Denmark), *Anas platyrhynchos*, *Anas poecilorhyncha*, *Anas rubripes*, *Anas strepera*	*Anas clypeata*, *Anas crecca*, *Anas penelope* (North America)
Diving ducks		*Aythya affinis*, *Aythya americana*, *Aythya ferina*, *Aythya marila*, *Aythya valisineria*, *Bucephala albeola*, *Bucephala clangula*, *Clangula hyemalis*, *Melanitta perspicillata*	*Melanitta deglandi*, *Oidemia americana*, *Oxyura jamaicensis*, *Polysticta stelleri*, *Somateria mollissima nigra*, *Somateria mollissima dresseri*, *Somateria spectabilis*
Geese	*Branta bernicla bernicla*, *Branta bernicla hrota*, *Branta bernicla nigricans*	*Branta canadensis*, *Chen canagica*	*Anser anser*
Swans	*Cygnus atratus*, *Cygnus olor*	*Cygnus cygnus*, *Cygnus buccinator *	*Cygnus columbianus*
Rails		*Fulica atra*	*Fulica americana*
Waders			*Limosa limosa islandica*


*Details underlying the categorization by importance in the diet are in **Supplementary Table S2**.*

## Discussion

Our review provides a quantitative perspective on the reciprocal linkages between waterfowl species and temperate seagrasses. The published literature provides multiple cases in which waterfowl abundances are closely linked to *Zostera* abundance, while also showing that herbivorous waterfowl can have strong top-down effects on *Zostera*. Of the 39 avian species documented to consume *Zostera*, only a small fraction (12 species) are represented in the studies suitable for inclusion in this meta-analysis. Below we discuss these results and reflect on how the effects of waterfowl herbivory could extend beyond direct consumption. We also discuss the implications of waterfowl–*Zostera* interactions for conservation and identify areas of future research.

### Reciprocal Linkages between Waterfowl and *Zostera*

The generally positive correlation between waterfowl abundance and *Zostera* abundance (**Figure 4** and **Supplementary Figure S2**) was expected and is consistent with predictive models (Stillman et al., 2015) and previous empirical studies. Ganter (2000) summarized historical reports of starving brant (*B. bernicla*) following dramatic losses of *Z. marina* from wasting disease along Atlantic shorelines in the 1930s. More recently, 95% losses of *Z. marina* from Antigonish Harbor, Nova Scotia, coincided with a 50% population decrease of Canada geese (*B. canadensis*) and goldeneyes (*Bucephala clangula*; Seymour et al., 2002). Pacific brant were notably reduced at their overwintering bays in Mexico in El Niño years, likely associated with decline in *Z. marina* under higher sea surface temperatures (Sedinger et al., 2006). Neither of these recent examples was included in the meta-analysis because they either had fewer than 4 years of data (Seymour et al., 2002) or a time series for only birds and not for *Zostera* (Sedinger et al., 2006). Our finding that the correlation between waterfowl and *Zostera* became more positive with increasing variation in *Zostera* (**Figure 4**) strengthens claims that waterfowl populations that utilize *Zostera* closely track the abundance of this resource over time and space. These results confirm that conservation of *Zostera* underpins management of many herbivorous waterfowl that either specialize on *Zostera* at particular seasonal stages or use it as high-quality forage.

The strong top-down effects of bird herbivory revealed in our meta-analysis (**Figure 5**) are consistent with several reports of over-consumption of seagrass, but not consistent with the generally low estimates of the fraction of *Zostera* entering the grazer food web. With respect to annual production, estimates of percent consumed by birds are generally low: e.g., 4% (Nienhuis and Groenendijk, 1986). However, estimates relative to standing biomass, winter productivity, or carrying capacity are frequently larger: 40% (Percival et al., 1998), 45% (Nacken and Reise, 2000), 50% (Jacobs et al., 1981; Baldwin and Lovvorn, 1994), 16–73% (Balsby et al., 2017), 80% (Hori and Hasegawa, 2005; Inger et al., 2006a), and total removal of 100% (Vermaat and Verhagen, 1996). Therefore, it appears possible for birds to have strong top-down effects seasonally even if this removal is a small fraction of total annual production, and reinforces that herbivory on *Zostera* by birds tends to be concentrated in time and space. These results contrast previous reviews of herbivory on marine primary producers (e.g., Cebrián and Duarte, 1998; Poore et al., 2012). Of the studies we included in our top-down analysis, only one (Portig et al., 1994) was also included in Poore et al. (2012). Furthermore, only six of the 193 studies in Poore et al. (2012) consider herbivory by birds, which suggests that interactions between birds and marine primary producers have not received the consideration of other herbivores like urchins, fishes, and crustaceans.

Any long-term loss of seagrass habitat can have consequences for a variety of avian herbivores (**Table 3**). Though the literature reports 39 species of avian consumers, this is likely an underestimate due to regional biases in the literature. Our search revealed proportionately more studies published on bird diets in Europe and North America (*n* = 29) compared to Asia (*n* = 1) or the southern hemisphere (*n* = 1). A number of waterfowl species (*n* > 5) feed on rhizomes and new shoots, which we expect to have a greater impact on growth and recovery of seagrass compared to consumption of *in situ* or detrital leaf matter. While not all examined waterfowl depend substantially on *Zostera*, these herbivores have the dietary flexibility to potentially have impacts on *Zostera* in a future with altered ranges or resource availabilities.

Although our study identified strong interactions between *Zostera* and waterfowl, our conclusions may not be generalizable to *Zostera* ecosystems worldwide. The genus *Zostera* has a near cosmopolitan distribution in temperate zones (**Figure 3**), and waterfowl (herbivorous or not) co-occur wherever *Zostera* grows. A lack of documentation at any one locale does not indicate that herbivory does not exist in that location. Large areas of the range of *Zostera* are poorly represented in our meta-analysis and in the peer-reviewed literature, such as Asia, the south Pacific, and Africa. On a smaller scale, many studies were done in specific locations because herbivory was observed there, resulting in a bias toward positive results in the studies selected for meta-analysis. Studies in which herbivory was not important may not have been published, or simply not done. Therefore, while our meta-analysis identified strong trends, the limited number and geographic spread of locations studied suggests that care must be taken in applying these conclusions more generally.

### Consequences of Herbivory for *Zostera* May Extend beyond Consumption

Potential connections between waterfowl and *Zostera* likely extend beyond the strong and direct consumptive effects shown in the meta-analysis and must be considered in assessing the full ecological impact of waterfowl on *Zostera* populations. Due to *Zostera*’s role as a foundation species, waterfowl have the potential to alter physiology, genetic diversity, phenology, dispersal, spatial dynamics, community composition, and ecosystem functioning.

#### Physiology

The loss of tissue to herbivory by definition reduces the biomass of the plant, but also can cause changes to plant physiology. Loss of leaf tissue can reduce growth rates, the production of side shoots, and can alter morphological traits (e.g., leaf length and width) of an individual shoot (as shown in clipping experiments: Ruesink et al., 2012; Hernan and Tomas, unpublished data; Kollars and Stachowicz, unpublished data). Damage by herbivores to the meristem or the rhizome leads to whole-shoot mortality and lowers shoot density within a meadow, and in occasional cases the bed can only recover densities because of high sexual reproduction (Kiriakopolos, 2013). Although compensatory growth following herbivory has been demonstrated in some tropical seagrasses (Valentine et al., 1997), it does not appear to be universal to all species (Cebrián and Duarte, 1998), has not been observed in clipping experiments in *Zostera* (Ruesink et al., 2012), and does not apply where a seagrass shoot is removed below the meristem.

#### Genetic Composition, Phenology, and Dispersal

Reductions of shoot density and canopy height within a meadow can act as a disturbance agent within *Zostera* populations. Disturbance may affect competition between seagrass clones and also alter the amount of resources (e.g., light) available for the recruitment of seedlings (e.g., Reusch, 2006). Herbivory might alter the genetic diversity of a seagrass population by selecting for genotypes with traits that are (1) resistant or resilient to the herbivore or (2) strong competitors or colonizers in post-disturbance recovery. Direct grazing on the area coverage of a seagrass population can indirectly alter genetic diversity by creating isolated patches that amplify genetic drift and/or affect gene flow by altering competition and resource sharing among genotypes with consequences to recruitment success. Therefore, genetic diversity can be both a product of grazing disturbance and a disturbance resilience mechanism (Hughes and Stachowicz, 2004; Hughes et al., 2007). These combined effects may lead to complex feedback loops between genetic diversity and realized disturbance severity.

Genetic composition of a population may also be altered by the effects of waterfowl on the life history and reproduction of seagrasses with consequential effects on recruitment dynamics. For example, heavy grazing by Canada geese (*B. canadensis*) in San Francisco, California has shifted the mating system of seagrass populations toward sexual reproduction over vegetative growth. This shift from low-herbivory perennial beds with clonal dynamics to high-herbivory annual beds dependent on the seed bank may eventually selectively favor early-flowering individuals (Kiriakopolos, 2013). Finally, waterfowl are potential dispersal agents of aquatic plants as seeds or rhizome fragments (first proposed in Darwin, 1859; McMahon et al., 2014). Sumoski and Orth (2012) showed that the lesser scaup (*Aythya affinis*) disperses *Z. marina* seeds via consumption and fecal deposition, and waterfowl may also be important in the dispersal of *Ruppia* spp. (Figuerola et al., 2003).

#### Spatial Structure

The environmental and landscape properties of seagrass beds may alter dynamics of bed persistence, faunal diversity, and ecosystem engineering (Boström et al., 2006). Depth and tidal pattern can determine whether seagrass is available to non-diving birds, which more heavily impact shallow and higher intertidal zones of the seagrass bed (Moore and Black, 2006). Digging behavior can also disrupt sediment and create gaps and hollows (Eklöf et al., 2011; Dos Santos et al., 2012) where loss of rhizomes may increase hydrodynamic disturbance and erosion (Eklöf et al., 2015). Interactions between seagrass, waterfowl, and other foundation species (e.g., lugworms in the Wadden Sea) can lead to complex spatial patterns within the seagrass bed that form from herbivory but are maintained by other ecosystem engineers (van der Heide et al., 2012). The interaction of grazing and sediment dynamics is not necessarily negative, however, and can have positive effects on the long-term persistence of seagrass beds. Nacken and Reise (2000) found that exclusion of birds from *Z. noltei* allowed higher shoot densities to persist through winter, but these high-density beds accumulated sediment that interfered with seagrass performance in the following growing season.

#### Associated Community and Ecosystem Function

Herbivory can affect the community composition and abundance of the flora and fauna associated with seagrass beds through increased patchiness (e.g., van der Heide et al., 2012), alterations to detrital pathways and food webs, and other mechanisms related to disturbance and canopy reduction. Analogous to the selective effects described for genetics and life history, herbivore preferences can affect macrophyte diversity by selecting for resistant or resilient species and altering competition among species. Herbivore-induced changes to shoot density, canopy height, morphology, and diversity can also alter the composition of the community associated with temperate seagrasses (e.g., Dixon, 2009; Eklöf et al., 2015), or population size and body mass in particular epifaunal species (Frimodig, 2007). Seagrass communities include taxa across the tree of life: bacteria, algae, infaunal invertebrates, epifaunal invertebrates, fishes, and waterfowl (Williams and Heck, 2001). Reduced canopy and increased patchiness alter predator–prey interactions by increasing visibility and thus risk for prey species (e.g., Hovel and Lipcius, 2001). Waterfowl herbivory may also alter the detrital food web. Direct consumption might reduce the productive biomass that becomes detritus, but inputs could instead be enhanced if grazers uproot shoots or dislodge leaves that are not consumed (i.e., sloppy feeding). Waterfowl fecal deposits can hypothetically be a source of nitrogen, though one experimental study found no effect of simulated grazing or fecal addition on community-level responses (Frimodig, 2007).

### Conservation Implications and Future Research

Although long-term losses due to grazing by waterfowl may be relatively infrequent (see Rivers and Short, 2007; Dos Santos et al., 2012), grazing can be of significant consequence for seagrass populations already in decline. Factors such as eutrophication, wasting disease, dredging and coastal development all contribute to reductions in *Zostera* populations worldwide (reviewed in Short and Wyllie-Echeverria, 1996; Orth et al., 2006). Furthermore, decreases in shoot density and rhizome mat integrity can threaten the overall survival of the bed. Therefore, destructive grazing that damages the rhizome may push *Zostera* beds beyond the point of shoot recovery (van der Heide et al., 2012; Eklöf et al., 2015).

Waterfowl whose ranges and population sizes have increased enough to be considered “nuisance species” have had severe and lasting impacts on *Zostera* beds due to overconsumption (e.g., Canada geese, Rivers and Short, 2007). The scope of our review did not allow us to evaluate conditions under which waterfowl become nuisance species, but we do echo other reports that overgrazing may result from the lessening of factors that limit bird population sizes (e.g., increased hunting restrictions, Bakker et al., 2016) and when bird taxa share little evolutionary history with seagrass populations (Wood et al., 2017). Some taxa, such as Canada geese, can live commensally with humans, which could expose seagrass habitats to their herbivory in coastal areas where other human activities have already degraded light or sediment conditions. As species introductions occur and ranges shift (Sorte et al., 2010; Vergés et al., 2014), the potential for unexpected interactions and impacts should be taken into account when considering management decisions.

The best studied non-native seagrass is *Z. japonica* in the northeastern Pacific (Harrison and Bigley, 1982; Williams, 2007; Shafer et al., 2013; Mach et al., 2014). Although *Z. japonica* habitat overlaps somewhat with *Z. marina*, *Z. japonica* grows higher on the shore and on hummocks (Nomme and Harrison, 1991; Hannam and Wyllie-Echeverria, 2015), and has generally come to dominate previously unvegetated intertidal zones (Mach et al., 2014). In our diet analysis (see **Supplementary Table S2**), we observed several cases in which *Z. japonica* is a novel food source for waterfowl species in this region, including those that do not depend on *Z. marina*. In Boundary Bay, British Columbia, *Z. japonica* composed the majority of the diet of the ducks *Anas acuta*, *Anas platyrhynchos*, the American widgeon *Anas americana*, and the Pacific brant *B. bernicla* (Baldwin and Lovvorn, 1994). *Z. marina* is a common dietary component of *A. americana* and *B. bernicla*, but is not a common food source for the other two ducks, indicating a novel use of seagrass. Canada geese (*B. canadensis*) also uproot whole *Z. japonica* shoots, eating only the meristems and generating detritus (Henry, personal observation). For species that commonly consume *Z. marina*, *Z. japonica* may have become a preferred food source because of its higher tidal elevation (and thus accessibility), small size (ease of handling) or potentially higher caloric value (Hori and Hasegawa, 2005). These novel interactions and uses by waterfowl are important to consider in decisions about the management and control of *Z. japonica*—decisions which vary by region (Shafer et al., 2013). Waterfowl have also been proposed as a potential propagule transport mechanism for *Z. japonica* (Shafer et al., 2013).

Predicting the consequences of *Zostera* loss on herbivorous waterfowl is more nuanced than understanding the effects of waterfowl on *Zostera* populations. Although our meta-analysis showed that bird populations track *Zostera* abundance, and in many cases result in real population declines (Seymour et al., 2002), there are numerous examples of birds shifting resource use to other locations or other forage, notably agricultural lands or macroalgae (Stewart, 1962; Ward et al., 2005). A recent study showed dietary shifts to agricultural lands to be beneficial for coastal birds (Fox and Abraham, 2017), as agricultural lands are a more consistent and nutritious food source than *Zostera*. Therefore, losses of *Zostera* may have the greatest consequence not for direct grazers, who may shift their feeding, but for those birds that depend on *Zostera* beds for habitat and to forage for epibiota living on the seagrass. For instance, great blue herons (*Ardea herodias*) forage disproportionately in *Zostera* habitats, which support high densities of fish (Huang et al., 2015; Gross et al., 2017).

For herbivorous birds, variability and losses of *Zostera* are likely to result in redistribution of bird populations and shifts in migration routes. Numerous shifts in bird ranges and migration routes have already been observed, either as direct responses to climate change or because climate stresses ecosystems in ways unsuitable for birds (Ward et al., 2005). Populations of both threatened and nuisance birds may increase and overtax resources (including *Zostera*) during their subsequent migration. Dietary shifts of geese to agricultural lands near their wintering grounds may result in better nutrition and higher reproductive success during the following breeding season (Fox and Abraham, 2017). In addition, many previously endangered waterfowl species are in recovery due to successful conservation efforts. Therefore, a population of birds that historically did not have long-term impacts on a *Zostera* bed may become problematic due to alterations to the population dynamics on the opposite side of their migration route. Understanding these linkages may require different protections and management strategies of bird populations as ranges and resource uses shift.

These conservation issues call for increased research efforts aimed at addressing the strength, context-dependency, and indirect effects of waterfowl–*Zostera* interactions in temperate systems. Despite the prevailing perspective that temperate seagrasses primarily fuel detrital food webs and are solely driven by bottom-up factors, reciprocal interactions between herbivorous birds and temperate seagrasses are well-documented worldwide. However, our ability to develop predictions of the reciprocal impacts between waterfowl and seagrasses is limited by the low number of hypothesis-driven experimental studies. Even though our meta-analyses identified multiple quantitative studies on waterfowl and *Zostera* interactions, only a handful of these studies involved the controlled manipulations necessary to assess reciprocal effects. Furthermore, most herbivory by waterfowl on temperate seagrasses occurs in the winter, a season that historically receives less scrutiny from field ecologists. Therefore, the seasonality of overlap between herbivorous birds and *Zostera* may hamper study, even as it decouples top-down effects from consumption as a fraction of annual production. Designing research programs that quantify the direct and indirect consequences of waterfowl–*Zostera* interactions across multiple spatio-temporal contexts will provide the data necessary to effectively inform management decisions on the joint conservation of both taxa.

## Author Contributions

All authors contributed to the development of ideas, collection of literature, and data extraction. CH and PJ provided unpublished data for inclusion in the meta-analyses. JR conducted the correlation meta-analysis, AH conducted the diet assessment, NK and MW conducted the top-down meta-analysis. NK, AH, MW, and JR co-wrote the manuscript with significant input from all authors.

## Conflict of Interest Statement

The authors declare that the research was conducted in the absence of any commercial or financial relationships that could be construed as a potential conflict of interest.

## Supplementary Material

**FIGURE S1 |** Funnel plot showing the relationship between the effect size and the standard error for the 14 studies used in the top-down meta-analysis.

**FIGURE S2 |** Relationships between bird and *Zostera* abundance. **(A)** Balsby et al. (2017), **(B)** Petersen et al. (2008), **(C)** Moore et al. (2004), **(D)** Wilson and Atkinson (1995), **(E)** Percival et al. (1998), **(F)** Tinkler et al. (2009), **(G)** Clausen and Percival (1998), **(H)** Dos Santos et al. (2012), **(I)** Dixon (2009). Data from Percival and Evans (1997) are presented in their Figure 4.

**TABLE S1 |** List and sources of references assessed for data suitable for meta-analyses, including diet, top down, and correlation between seagrass and bird populations. Studies noted as “Not applied” are those that did not have data meeting the criteria for analyses presented in this paper, but were relevant.

**TABLE S2 |** Bird species identified as consuming *Zostera* and summary of evidence to support *Zostera* consumption, including observational studies and diet analyses.
