# Supplementary figures and images for: Meta-Analysis of Reciprocal Linkages between Temperate Seagrasses and Waterfowl with Implications for Conservation
Source: Front Plant Sci. 2017 Dec 22;8:2119. doi: 10.3389/fpls.2017.02119 (PMC5744074; doi:10.3389/fpls.2017.02119)

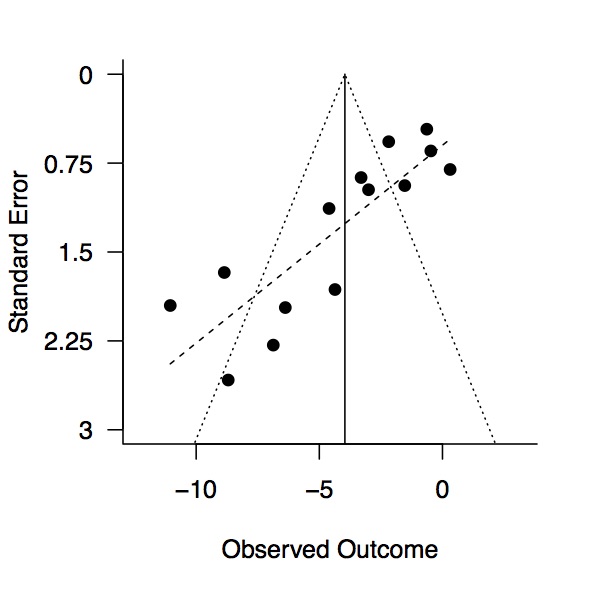

Supplement: Supplementary file 1 [file Image_1.jpeg]

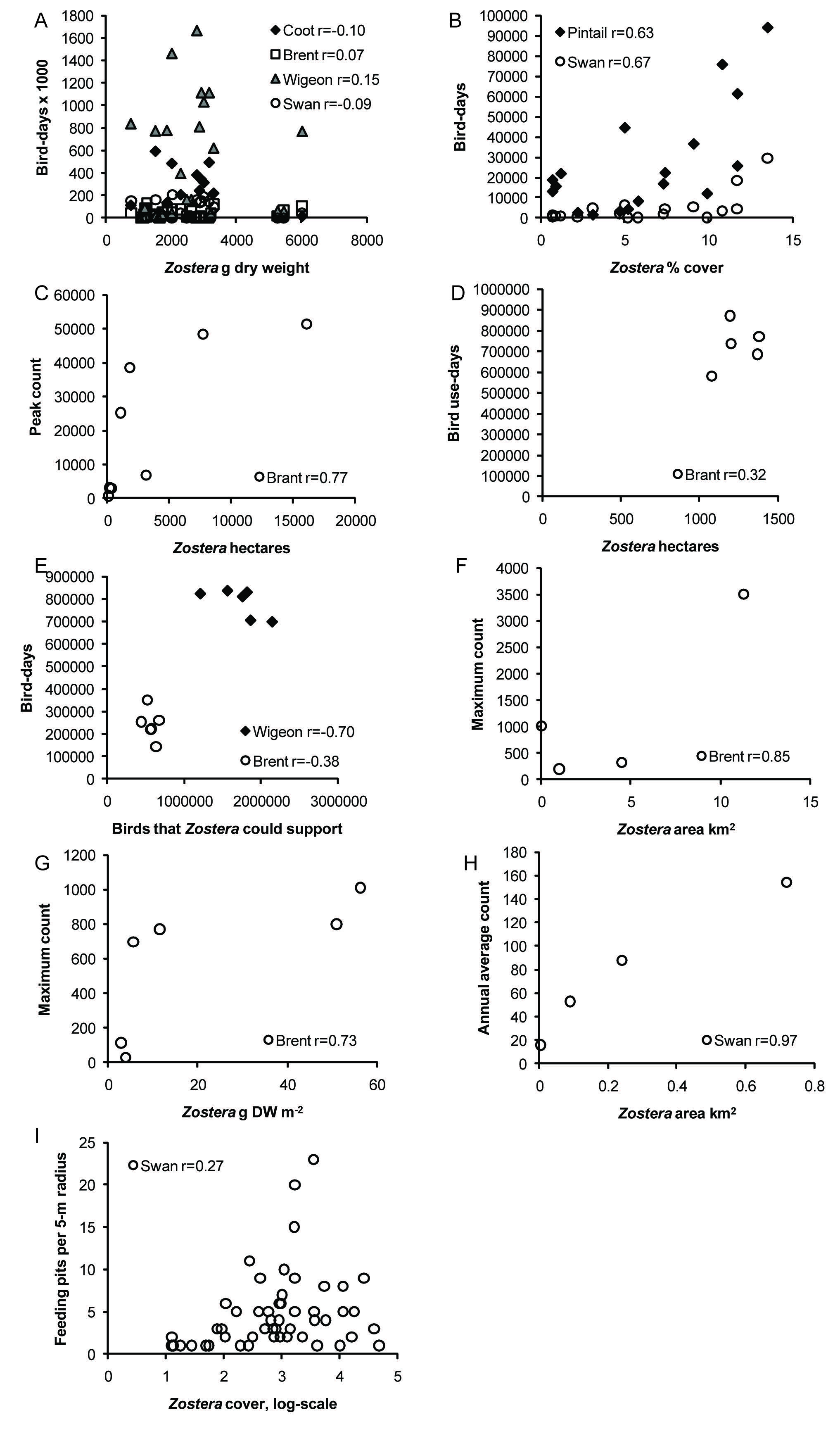

Supplement: Supplementary file 2 [file Image_2.jpeg]
