# Supplementary material for: Meta-Analysis of Reciprocal Linkages between Temperate Seagrasses and Waterfowl with Implications for Conservation
Source: Front Plant Sci. 2017 Dec 22;8:2119. doi: 10.3389/fpls.2017.02119 (PMC5744074; doi:10.3389/fpls.2017.02119)
Supplement: Supplementary file 3 [file Table_1.PDF]

Table S1: List and sources of references assessed for data suitable for meta-analyses, including diet, top down, and correlation between seagrass and bird populations. Studies noted as "Not applied" are those that did not have data meeting the criteria for analyses presented in this paper, but were relevant.

| Reference | Analysis | Source |
|---|---|---|
| Baldwin, J.R. and Lovvorn, J.R. (1994) Expansion of seagrass habitat by the exotic *Zostera japonica*, and its use by dabbling ducks and brant in Boundary Bay, British Columbia. *Mar Ecol Prog Ser* 103, 119-127. | Diet | 15 Nov 2016 Web of Science: (Zostera and ((bird* and (herbiv* or graz*)) or (waterfowl or goose or geese or brant)) |
| Baldwin, J.R. and Lovvorn, J.R. (1994). Habitats and tidal accessibility of the marine foods of dabbling ducks and brant in Boundary Bay, British Columbia. Marine Biology 120, 627-638. | Not applied | 15 Nov 2016 Web of Science: (Zostera and ((bird* and (herbiv* or graz*)) or (waterfowl or goose or geese or brant)) |
| Balsby, T. J. S., Clausen, P., Krause-Jensen, D., Carstensen, J., and Madsen, J. (2017) Long-term patterns of eelgrass (*Zostera marina*) occurrence and associated herbivorous waterbirds in a Danish coastal inlet. *Frontiers in Marine Science* 3(285), 1-14. | Correlation, Diet | Pers. comm. P. Clausen |
| Campbell, J. W. 1946. The Food of the Wigeon and Brent Goose. *British Birds* 39, 194-200. | Diet | Cited by Charman 1977 |
| Charman, K. (1977). The grazing of *Zostera* by waterfowl. *Aquaculture* 12, 229-233. | Diet | Cited by Tinkler et al. 2009 |
| Clausen P. (2000). Modelling water level influence on habitat choice and food availability for *Zostera* feeding brent geese *Branta bernicla* in non-tidal areas. *Wildlife Biology* 6, 75-87. | Not applied | 15 Nov 2016 Web of Science: (Zostera and ((bird* and (herbiv* or graz*)) or (waterfowl or goose or geese or brant)) |

| | | |
|---|---|---|
| Clausen, K. K., and Clausen, P. (2014). Forecasting future drowning of coastal waterbird habitats reveals a major conservation concern. *Biological Conservation* 171, 177-185. | Not applied | 15 Nov 2016 Web of Science: (Zostera and ((bird* and (herbiv* or graz*)) or (waterfowl or goose or geese or brant)) |
| Clausen, K. K., Clausen, P., Faelled, C. C., and Mouritsen, K. N. (2012). Energetic consequences of a major change in habitat use: endangered Brent Geese *Branta bernicla hrota* losing their main food resource. *Ibis* 154, 803-814. | Diet | 15 Nov 2016 Web of Science: (Zostera and ((bird* and (herbiv* or graz*)) or (waterfowl or goose or geese or brant)) |
| Clausen, P. (1994). On the impact of spring grazing by brent geese on *Zostera marina* growth, reproduction, and availability as a food resource. In: Waterfowl as primary consumers in shallow water fjord areas. PhD thesis, University of Aarhus/National Environmental Research Institute, Denmark, 42-55. | Not applied | Pers. comm. P. Clausen |
| Clausen, P. and Percival, S. M. (1998). Changes in distribution and habitat use of Svalbard light-bellied brent geese *Branta bernicla hrota*, 1980-1995: Driven by *Zostera* availability? Pp. 253-276 in Mehlum, F., Black, J. M., and Madsen, J. (eds.) Research on Arctic Geese. Proceedings of the Svalbard Goose Symposium, Oslo, Norway, 23-26 September 1997. *Norsk Polarinstitutt Skrifter* 200. | Correlation | 15 Nov 2016 Web of Science: (Zostera and ((bird* and (herbiv* or graz*)) or (waterfowl or goose or geese or brant)) |
| Clausen, P., Madsen, J., Percival, S. M., O'Connor, D., and Anderson, G. Q. A. (1998). Population development and changes in winter site use by the Svalbard light-bellied brent goose, *Branta bernicla hrota 1980-1994. Biological Conservation* 84, 157-165. | Not applied | 15 Nov 2016 Web of Science: (Zostera and ((bird* and (herbiv* or graz*)) or (waterfowl or goose or geese or brant)) |

| | | |
|---|---|---|
| Collinge, W. E. (1936). II.—The Food and Feeding-habits of the Coot (*Fulica atra* Linn.). *Ibis* 78, 35–39. doi: 10.1111/j.1474-919X.1936.tb03646.x | Diet | 15 Aug 2017 Web of Science: (Zostera and (coot or Fulica)) |
| Cottam C. (1939). Food habits of North American diving ducks. US Department of Agriculture. Technical Bulletin 643. | Diet | Cited in Olsen 2015 |
| Desmonts, D., Fritz, H., Cornulier, T., and Maheo, R. (2009). Rise in human activities on the mudflats and Brent Geese (*Branta bernicla*) wintering distribution in relation to *Zostera* spp. beds: a 30-year study. *Journal of Ornithology* 150, 733-742. | Not applied | 15 Nov 2016 Web of Science: (Zostera and ((bird* and (herbiv* or graz*)) or (waterfowl or goose or geese or brant)) |
| Dixon, H. D. J. (2009). Effect of black swan foraging on seagrass and benthic invertebrates in western Golden Bay. MSc Thesis. Massey University, Palmerston North, New Zealand. | Correlation, Top down, Diet | Cited by Wood et al. 2012 |
| Dos Santos, V. M., Matheson, F. E., Pilditch, C. A., and Elger, A. (2012). Is black swan grazing a threat to seagrass? Indications from an observational study in New Zealand. *Aquatic Botany* 100, 41-50. | Correlation, Top down Diet | 15 Nov 2016 Web of Science: (Zostera and ((bird* and (herbiv* or graz*)) or (waterfowl or goose or geese or brant)) |
| Eklof, J. S., van der Heide, T., Donadi, S., van der Zee, E. M., O'Hara, R., and Eriksson, B. K. (2011) Habitat-mediated facilitation and counteracting ecosystem engineering interactively influence ecosystem responses to disturbance. *PLoS One* 6(8), e23229 | Top down | 15 Nov 2016 Web of Science: (Zostera and ((bird* and (herbiv* or graz*)) or (waterfowl or goose or geese or brant)) |
| Fox, A. D. (1996). *Zostera* exploitation by Brent geese and wigeon on the Exe estuary, southern England. *Bird Study* 43, 257-268. | Not applied | 15 Nov 2016 Web of Science: (Zostera and ((bird* and (herbiv* or graz*)) or (waterfowl or goose or geese or brant)) |

| | | |
|---|---|---|
| Frazier, M. R., Lamberson, J. O., and Nelson, W. G. (2014). Intertidal habitat utilization patterns of birds in a Northeast Pacific estuary. *Wetlands Ecology and Management* 22:451-466. | Not applied | 15 Nov 2016 Web of Science: (Zostera and ((bird* and (herbiv* or graz*)) or (waterfowl or goose or geese or brant)) |
| Ganter, B. (2000). Seagrass (*Zostera* spp) as food for brent geese (*Branta bernicla*): an overview. *Helogland Mar Res* 54, 63-70. | Diet | 15 Nov 2016 Web of Science: (Zostera and ((bird* and (herbiv* or graz*)) or (waterfowl or goose or geese or brant)) |
| Gayet, G., Croce, N., Grillas, P., Nourry, C., Deschamps, C., and Defos du Rau, P. (2012). Expected and unexpected effects of waterbirds on Mediterranean aquatic plants. *Aquatic Botany* 103, 98-105. | Top down, Diet | 15 Nov 2016 Web of Science: (Zostera and ((bird* and (herbiv* or graz*)) or (waterfowl or goose or geese or brant)) |
| Headley, P.C. (1966). Ecology of the emperor goose. Alaska Cooperative Wildlife Unit. Wildlife Research Unit Studies VII. | Diet | Cited in Olsen 2015 |
| Hori, M., and Hasegawa, N. (2005). Consumption of benthic organisms by birds in coastal ecosystems. *Japanese Journal of Benthology* 60, 12–22. http://doi.org/10.1016/j.aquabot.2006.12.002 | Diet | Pers. Comm. authors |
| Hughes, A. R., and Stachowicz, J. J. (2004). Genetic diversity enhances the resistance of a seagrass ecosystem to disturbance. *Proc Nat Acad Sci USA* 101, 8998-9002. | Not applied | 15 Nov 2016 Web of Science: (Zostera and ((bird* and (herbiv* or graz*)) or (waterfowl or goose or geese or brant)) |
| Inger, R., Ruxton, G. D., Newton, J., Colhoun, K., Mackie, K., Robinson, J. A., and Bearhop, S. (2006). Using daily ration models and stable isotope analysis to predict biomass depletionby herbivores. *J Appl Ecol* 43, 1022-1030. | Not applied | 15 Nov 2016 Web of Science: (Zostera and ((bird* and (herbiv* or graz*)) or (waterfowl or goose or geese or brant)) |

| | | |
|---|---|---|
| Inger, R., Ruxton, G. D., Newton, J., Colhoun, K., Mackie, K., Robinson, J. A., Jackson, A. L., and Bearhop, S. (2006). Temporal and intrapopulation variation in prey choice of wintering geese determined by stable isotope analysis. *J Anim Ecol* 75, 1190-1200. | Not applied | 15 Nov 2016 Web of Science: (Zostera and ((bird* and (herbiv* or graz*)) or (waterfowl or goose or geese or brant)) |
| Jacobs, R. P. W. M., den Hartog, C., Braster, B.F. and Carriere, F. C. (1981). Grazing of the seagrass *Zostera noltii* by birds at Terschelling (Dutch Wadden Sea) *Aquat Bot* 10, 241-259. | Top down, diet | 15 Nov 2016 Web of Science: (Zostera and ((bird* and (herbiv* or graz*)) or (waterfowl or goose or geese or brant)) |
| Johnsgard, P. A. (1978). Ducks, geese, and swans of the world. University of Nebraska Press, Lincoln. | Diet | Cited in Olsen 2015 |
| Jones, J. C. (1940). Food habits of the American Coot with notes on distribution. Bureau of Biological Survey, U.S. Department of the Interior. Wildlife Research Bulletin 2. Washington. | Diet | 15 Aug 2017 Web of Science: (Zostera and (coot or Fulica)) |
| Ladin, Z. S., Castelli, P. M., Mcwilliams, S. R., and Williams, C. K. (2011). Time energy budgets and food use of Atlantic Brant across their wintering range. *J Wildlife Management* 75, 273-282. | Diet | 15 Nov 2016 Web of Science: (Zostera and ((bird* and (herbiv* or graz*)) or (waterfowl or goose or geese or brant)) |
| Lamberson, J. O., Frazier, M. R., Nelson, W. G., and Clinton, P. J. (2011). Utilization Patters of Intertidal Habitats by Birds in Yaquina Estuary, Oregon. U.S. Environmental Protection Agency, Washington, DC, EPA/600/R-11/118 (NTIS PB2012-110757). | Diet | Cited in Shafer et al. 2013 |

| | | |
|---|---|---|
| Lopez-Calderon, J. M., Riosmena-Rodriguez, R., Torre, J., Meling, A., and Basurto, X. (2016). *Zostera marina* meadows from the Gulf of California: conservation status. *Biodiversity and Conservation* 25, 261-273. | Not applied | 15 Nov 2016 Web of Science: (Zostera and ((bird* and (herbiv* or graz*)) or (waterfowl or goose or geese or brant)) |
| Lovvorn, J. R. and Baldwin, J.R. (1996). Intertidal and farmland habitats of ducks in the Puget Sound region: a landscape perspective. *Biological Conservation* 77, 97-114. | Diet | 15 Nov 2016 Web of Science: (Zostera and ((bird* and (herbiv* or graz*)) or (waterfowl or goose or geese or brant)) |
| Madsen J. (1988). Autumn feeding ecology in the Danish Wadden Sea, and impact of food supplies and shooting on movement. *Dan Rev Game Biol* 13, 1-32. | Top Down | Valentine and Heck 1999 |
| Martinez Cedillo, I., Carmona, R., Ward, D. H., and Danemann, G. D. (2013). Habitat use patterns of the Black Brant *Branta bernicla nigricans* (Anseriformes: Anatidae) in natural and artificial areas of Guerrero Negro, Baja California Sur, Mexico. *Revista de Biologia Tropical* 61, 927-935. | Not applied | 15 Nov 2016 Web of Science: (Zostera and ((bird* and (herbiv* or graz*)) or (waterfowl or goose or geese or brant)) |
| Mathers, R. G., Montgomery, W.I., and Portig, A. A. (1998). Exploitation of intertidal *Zostera* species by Brent geese (*Branta bernicla hrota*): Why dig for your dinner? *Biology and Environment Proc Roy Irish Acad* 98B, 147-152 and 99B, 171. | Not applied | 15 Nov 2016 Web of Science: (Zostera and ((bird* and (herbiv* or graz*)) or (waterfowl or goose or geese or brant)) |
| Mathers, R.G., and Montgomery, W. I. (1996). Behaviour of Brent geese *Branta bernicla hrota* and wigeon *Anas penelope* feeding on intertidal *Zostera* spp. *Biology and Environment - Proc Roy Irish Acad* 96B, 159-167. | Diet | 15 Nov 2016 Web of Science: (Zostera and ((bird* and (herbiv* or graz*)) or (waterfowl or goose or geese or brant)) |

| | | |
|---|---|---|
| Mathers, R.G., Portig, A.A., and Montgomery, W.I. (1998). Distribution and abundance of Pale-bellied Brent Geese and Wigeon on Strangford Lough, Northern Ireland. *Bird Study* 45, 18-34. | Not applied | 15 Nov 2016 Web of Science: (Zostera and ((bird* and (herbiv* or graz*)) or (waterfowl or goose or geese or brant)) |
| Mathiasson S. (1973). A moulting population of non-breeding Mute Swans with special reference to flight-feather moult, feeding ecology, and habitat selection. *Wildfowl* 24, 43-53. | Diet | Cited in Olsen 2015 |
| McConnaughey T., and McRoy C.P. (1979). 13C label identifies eelgrass (*Zostera marina*) carbon in an Alaskan estuarine food web. *Mar Biol* 53, 263-269. | Not applied | Cited in Valentine and Heck 1999 |
| McKelvey, R.W. (1981). Some aspects of the winter feeding ecology of trumpeter swans at Port Alberni and Comox Harbor, British Columbia. Msc Thesis. Simon Fraser University, Vancouver, BC. | Diet | Cited in Olsen 2015 |
| McMahan, C. A. (1970). Food Habits of Ducks Wintering on Laguna Madre, Texas. *The Journal of Wildlife Management*, 34(4), 946. http://doi.org/10.2307/3799167 | Not Applied | Cited in Olsen 2015 |
| McRoy, C.P. (1966) The standing stock and ecology of eelgrass (*Zostera marina* L.) in Izembek Lagoon, Alaska. MS thesis. University of Washington, Seattle, WA. | Not applied | Cited in Baldwin & Lovvorn 1994 |
| Moore, J.E. and Black, J.M. 2006. Slave to the tides: Spatiotemporal foraging dynamics of spring staging Black Brant. *Condor* 108, 661-677. | Not applied | 15 Nov 2016 Web of Science: (Zostera and ((bird* and (herbiv* or graz*)) or (waterfowl or goose or geese or brant)) |

| | | |
|---|---|---|
| Moore, J.E., and Black, J.M. (2006). Historical changes in brant *Branta bernicla nigricans* use on Humboldt Bay, California. *Wildlife Biology* 12, 151-162. | Not applied | 15 Nov 2016 Web of Science: (Zostera and ((bird* and (herbiv* or graz*)) or (waterfowl or goose or geese or brant)) |
| Moore, J.E., Colwell, M.A., Mathis, R. L., and Black, J. M. (2004). Staging of Pacific flyway brant in relation to eelgrass abundance and site isolation, with special consideration of Humboldt Bay, California. *Biological Conservation* 115, 475-486. | Correlation | 15 Nov 2016 Web of Science: (Zostera and ((bird* and (herbiv* or graz*)) or (waterfowl or goose or geese or brant)) |
| Nacken, N. and Reise, K. (2000). Effects of herbivorous birds on intertidal seagrass beds in the northern Wadden Sea. *Helg Mar Res* 54, 87-94. | Not applied | 15 Nov 2016 Web of Science: (Zostera and ((bird* and (herbiv* or graz*)) or (waterfowl or goose or geese or brant)) |
| Nienhuis, P.H. (1993). Nutrient cycling and foodwebs in Dutch estuaries. *Hydrobiologia* 265, 15-44. | Not applied | 15 Nov 2016 Web of Science: (Zostera and ((bird* and (herbiv* or graz*)) or (waterfowl or goose or geese or brant)) |
| Nienhuis, P.H., and Groenendijk, A.M. (1986). Consumption of eelgrass (*Zostera marina)* by birds and invertebrates: an annual budget. *Mar Ecol Prog Ser* 29, 29-35. | Diet | Cited in Valentine and Heck 1999 |
| Nienhuis, P.H., and van Ierland, E.T. (1978). Consumption of eelgrass, *Zostera marina,* by birds and invertebrates during the growing season in Lake Grevekingen (SW Netherlands). *Neth J Sea Res* 12, 180-194. | Diet | Cited in Baldwin and Lovvorn 1994 |
| Olsen, A. M. (2015). Exceptional avian herbivores: multiple transitions toward herbivory in the bird order Anseriformes and its correlation with body mass. *Ecology and Evolution*, *5*(21), 5016–5032. http://doi.org/10.1002/ece3.1787 | Not applied | pers. comm. A. Olsen, to Amy Henry |

| | | |
|---|---|---|
| Percival, S. (1991). Durham University project on the feeding ecology of Brent geese and Wigeon at Lindisfarne NNR. Progress report to the Lindisfarne Wildfowling Panel, pp. 1-2 {In Portig 1994} | Not applied | 15 Nov 2016 Web of Science: (Zostera and ((bird* and (herbiv* or graz*)) or (waterfowl or goose or geese or brant)) |
| Percival, S.M., and Evans, P.R. (1997). Brent Geese *Branta bernicla* and *Zostera*; Factors affecting the exploitation of a seasonally declining food resource. *Ibis* 139, 121-128. | Correlation | 15 Nov 2016 Web of Science: (Zostera and ((bird* and (herbiv* or graz*)) or (waterfowl or goose or geese or brant)) |
| Percival, S.M., Sutherland, W.J., and Evans, P.R. (1996). A spatial depletion model of the responses of grazing wildfowl to the availability of intertidal vegetation. *J Applied Ecology* 33, 979-992 | Not applied | 15 Nov 2016 Web of Science: (Zostera and ((bird* and (herbiv* or graz*)) or (waterfowl or goose or geese or brant)) |
| Percival, S.M., Sutherland, W.J., and Evans, P.R. (1998). Intertidal habitat loss and wildfowl numbers: applications of a spatial depletion model. *J Applied Ecology* 35, 57-63. | Correlation | 15 Nov 2016 Web of Science: (Zostera and ((bird* and (herbiv* or graz*)) or (waterfowl or goose or geese or brant)) |
| Petersen, J. K., Hansen, J.W., Baursen, M. B., Clausen, P., Carstensen, J., and Conley, D. J. 2008. Regime shift in a coastal marine ecosystem. *Ecol Appl* 18, 497-510. | Correlation | Pers. comm. P. Clausen |
| Petrich, C., Tivy, A. C., and Ward, D. H. (2014). Reconstruction of historic sea ice conditions in a sub-Arctic lagoon. *Cold Regions Science and Technology* 98, 55-62. | Not applied | 15 Nov 2016 Web of Science: (Zostera and ((bird* and (herbiv* or graz*)) or (waterfowl or goose or geese or brant)) |
| Pettifor, R.A., Caldow, R. W. G., Rowcliffe, J. M., Goss-Custard, J. D., Black, J.M., Hodder, K.H., Houston, A. I., Lang A., and Webb, J. (2000). Spatially explicit, individual-based, behavioural models of the annual cycle of two migratory goose populations. *J Applied Ecology* 37, 103-135. | Not applied | Cited by Shaugnessy et al. 2012 |

| | | |
|---|---|---|
| Ponsero, A., Le Mao, P., Yesou, P., Jeremy, A., and Vidal, J. (2009). Ecosystem quality and natural heritage preservation: the case of the littoral eutrophication and the wintering of Brent Geese *Branta b. bernicla* in the bay of Saint-Brieuc (France). *Revue d'Ecologie - La terre et la vie* 64, 157-170. | Not applied | 15 Nov 2016 Web of Science: (Zostera and ((bird* and (herbiv* or graz*)) or (waterfowl or goose or geese or brant)) |
| Portig, A. A., Mathers, R. G., Montgomery, W. I., and Govier, R. N. (1994). The distribution and utilisation of *Zostera* species in Strangford Lough, Northern Ireland. *Aquat Bot* 47, 317-328. | Not applied | Cited by Valentine and Heck 1999 |
| Ranwell, D. S., and Downing, B. M. (1959). Brent goose (*Branta bernicula (L.)*) winter feeding pattern and *Zostera* resources at Scolt Head Island, Norfolk. *Animal Behavior* 7(1-2), 42-56. | Diet | Cited by Baldwin & Lovvorn 1994 |
| Rivers, D. O. and Short, F. T. (2007). Effect of grazing by Canada geese *Branta canadensis* on an intertidal eelgrass *Zostera marina* meadow. *Mar Ecol Prog Ser* 333, 271-279. | Top down, Diet | 15 Nov 2016 Web of Science: (Zostera and ((bird* and (herbiv* or graz*)) or (waterfowl or goose or geese or brant)) |
| Robin, F., Piersma, T., Meunier, F., and Bocher, P. (2013). Expansion into an herbivorous niche by a customary carnivore: black-tailed godwits feeding on rhizomes of *Zostera* at a newly established wintering site. *Condor* 115, 340-347. | Diet | 15 Nov 2016 Web of Science: (Zostera and ((bird* and (herbiv* or graz*)) or (waterfowl or goose or geese or brant)) |

| | | |
|---|---|---|
| Robinson, J.A., Colhoun, K., Gudmundsson, G.A., Boertmann, D., Merne, O.J., O'Brian, M., Portig, A.A., Mackie, K., and Boyd, H. (2004). Light-bellied Brent Goose *Branta bernicla hrota* (East Canadian High Arctic population) in Canada, Ireland, Iceland, France, Greenland, Scotland, Wales, England, the Channel Islands and Spain 1960/61 – 1999/2000. *Waterbird Review Series.* The Wildfowl & Wetlands Trust/Joint Nature Conservation Committee, Slimbridge. | Diet | Cited in Clausen et al. 2012 |
| Rodewald, P. (Editor). (2015). The Birds of North America. https://birdsna.org. Cornell Laboratory of Ornithology, Ithaca, NY. | Diet | Cited in Olsen 2015 |
| Schamber, J. L., Sedinger, J. S., and Ward, D.H. (2012). Carry-over effects of winter location contribute to variation in timing of nest initiation and clutch size in Black Brant (*Branta bernicla nigricans*). *Auk* 129, 205-210. | Not applied | 15 Nov 2016 Web of Science: (Zostera and ((bird* and (herbiv* or graz*)) or (waterfowl or goose or geese or brant)) |
| Sedinger, J. S., Schamber, J. L., Ward, D. H., Nicolai, C. A., and Conant, B. (2011). Carryover effects associated with winter location affect fitness, social status, and population dynamics of a long-distance migrant. *Am Nat* 178, E110-E123. | Not applied | 15 Nov 2016 Web of Science: (Zostera and ((bird* and (herbiv* or graz*)) or (waterfowl or goose or geese or brant)) |
| Sedinger, J.S., Ward, D.H., Schamber, J.L., Butler, W. I., Eldridge, W. D., Conant, B., Voelzer, J. F., Chelgren, N. D., and Herzog, M. P. (2006). Effects of El Nino on distribution and reproductive performance of Black Brant. *Ecology* 87:151-159. | Not applied | 15 Nov 2016 Web of Science: (Zostera and ((bird* and (herbiv* or graz*)) or (waterfowl or goose or geese or brant)) |

| | | |
|---|---|---|
| Seymour, N.R., Miller, A.G., and Garbary, D.J. (2002). Decline of Canada geese (*Branta canadensis*) and common goldeneye (*Bucephala clangula*) associated with a collapse of eelgrass (*Zostera marina*) in a Nova Scotia estuary. *Helgoland Mar Res* 56, 198-202. | Diet | 15 Nov 2016 Web of Science: (Zostera and ((bird* and (herbiv* or graz*)) or (waterfowl or goose or geese or brant)) |
| Shaughnessy, F. J., Gilkerson, W., Black, J. M., Ward, D. H., and Petrie, M. (2012). Predicted eelgrass response to sea level rise and its availability to foraging Black Brant in Pacific coast estuaries. *Ecological Applications* 22(6), 1743-1761. | Not applied | 15 Nov 2016 Web of Science: (Zostera and ((bird* and (herbiv* or graz*)) or (waterfowl or goose or geese or brant)) |
| Stahl, J., Bos, D., and Loonen, M.J.J.E. (2002). Foraging along a salinity gradient - The effect of tidal inundation on site choice by Dark-Bellied Brent Geese *Branta bernicla* and Barnacle Geese *B. leucopsis*. *Ardea* 90, 201-212. | Not applied | 15 Nov 2016 Web of Science: (Zostera and ((bird* and (herbiv* or graz*)) or (waterfowl or goose or geese or brant)) |
| Stewart, R. E. (1962). Waterfowl populations in the upper Chesapeake region. U.S. Fish Wildl. Serv. Spec. Sci. Rep.: Wildl. no. 65. | Diet | Cited in Olsen 2015 |
| Stillman, R. A., Wood, K. A., Gilkerson, W., Elkinton, E., Black, J. M., Ward, D. H., and Petrie, M. (2015). Predicting effects of environmental change on a migratory herbivore. *Ecosphere* 6(7), 114. | Not applied | 15 Nov 2016 Web of Science: (Zostera and ((bird* and (herbiv* or graz*)) or (waterfowl or goose or geese or brant)) |
| Tinkler, E., Montgomery, W. I., and Elwood, R. W. (2009). Foraging ecology, fluctuating food availability and energetics of wintering brent geese. *J Zoology* 278, 313-323. | Correlation, Top down | 15 Nov 2016 Web of Science: (Zostera and ((bird* and (herbiv* or graz*)) or (waterfowl or goose or geese or brant)) |

| | | |
|---|---|---|
| Tubbs C.R., and Tubbs J.M. (1983). The distribution of *Zostera* and its exploitation by wildfowl in the Solent, Southern England. *Aquat Bot* 15, 223-239. | Top down | Cited in Valentine and Heck 1999 |
| van der Heide, T., Eklof, J. S., van Nes, E. H., van der Zee, E. M., Donadi, S., Weerman, E. J., Olff, H., and Eriksson, B. K. (2012). Ecosystem Engineering by Seagrasses Interacts with Grazing to Shape an Intertidal Landscape. *PLoS One* 7(8), e42060. | Not applied | 15 Nov 2016 Web of Science: (Zostera and ((bird* and (herbiv* or graz*)) or (waterfowl or goose or geese or brant)) |
| Vermaat, J.E., and Verhagen, F.C.A. (1996). Seasonal variation in the intertidal seagrass *Zostera noltii* Hornem: coupling demographic and physiological patterns. *Aquat Bot* 52, 259-281. | Not applied | 15 Nov 2016 Web of Science: (Zostera and ((bird* and (herbiv* or graz*)) or (waterfowl or goose or geese or brant)) |
| Ward, D.H. (1983) The relationship of two seagrasses *Zostera marina* and *Ruppia maritima* to the black brant, *Branta bernicla nigricans*, San Ignacio Lagoon, Baja California, Mexico. MS thesis. University of Oregon, Eugene, OR. | Not applied | Cited in Baldwin and Lovvorn 1994 |
| Ward, D.H., Reed, A., Sedinger, J. S., Black, J. M., Derksen, D. V., and Castelli, P. M. (2005). North American Brant: effects of changes in habitat and climate on population dynamics. *Global Change Biology* 11, 869-880. | Not applied | 15 Nov 2016 Web of Science: (Zostera and ((bird* and (herbiv* or graz*)) or (waterfowl or goose or geese or brant)) |
| Wilkins, E.W. (1982). Waterfowl utilization of a submerged vegetation (*Zostera marina* and *Rupplia maritima*) bed in the lower Chesapeake Bay. MSc thesis. College of William and Mary, Williamsburg, VA. | Not applied | Cited in Baldwin and Lovvorn 1994 |

| | | |
|---|---|---|
| Wilson, U.W., and Atkinson, J. B. (1995). Black brant winter and spring-staging use at two Washington coastal areas in relation to eelgrass abundance. *Condor* 97, 91-98. | Correlation | 15 Nov 2016 Web of Science: (Zostera and ((bird* and (herbiv* or graz*)) or (waterfowl or goose or geese or brant)) |
| Wyer, D. W., Boorman, L. A., and Waters, R. (1977). Studies on the distribution of *Zostera* in the outer Thames estuary. *Aquaculture* 12, 215-227. | Not applied | Cited in Tubbs and Tubbs 1983 |
| Yocum, C. F., and Keller, M. (1961). Correlation of food habits and abundance of waterfowl, Humboldt Bay, California. *California Fish and Game* 47, 41-53. | Diet | Cited in Olsen 2015 |
