# Supplementary material for: Meta-Analysis of Reciprocal Linkages between Temperate Seagrasses and Waterfowl with Implications for Conservation
Source: Front Plant Sci. 2017 Dec 22;8:2119. doi: 10.3389/fpls.2017.02119 (PMC5744074; doi:10.3389/fpls.2017.02119)
Supplement: Supplementary file 4 [file Table_2.PDF]

Table S2: Bird species identified as consuming *Zostera* and summary of evidence to support *Zostera* consumption, including observational studies and diet analyses.

Species name, common name, type	Summary of diet studies and observations	References
Anas acuta, Northern pintail, dabbling duck	In Boundary Bay, BC, *Z. japonica* leaves, (8%), seeds (16%) and rhizomes (24%) made up half of esophageal contents in a sample of 54 ducks (Baldwin and Lovvorn 1994), with *Z. marina* leaves 3%. In Terschelling (Jacobs 1981), wintering birds removed 26% of the annual production of *Z. noltei* in 1974 (50% of standing stock), and *A. acuta* was estimated responsible for 20% of the biomass removed. In Lake Akkeshi, Japan, they were observed eating *Z. marina* and *Z. japonica*, with highest consumption in autumn (Hori and Hasegawa 2005). In the Netherlands (Nienhuis and Van Ierland 1978), *A. acuta* is a rare consumer of eelgrass, but estimated to constitute 25% of its diet (Nienhuis and Groenendijk 1978).	Nienhuis and Van Ierland 1978, Nienhuis and Groenendijk 1986, Jacobs et al 1981, Baldwin and Lovvorn 1994, Lovvorn and Baldwin 1996, Hori and Hasegawa 2005
Anas americana, American wigeon, dabbling duck	In Boundary Bay, BC, *Z. japonica* leaves, (84%), and rhizomes (~1%) made up the majority of esophageal contents in a sample of 45 widgeons (Baldwin and Lovvorn 1994), while *Z. marina* leaves were 3%. Widgeon were estimated to get 43% of their food from intertidal zones, though not exclusively on *Zostera*. In Humboldt Bay, CA, (Yocum and Keller 1961) *Z. marina* composed 81% of diet in 1961. In Chesapeake Bay (Stewart 1962), *Z. marina* was 30-67% (mean 53%) of gut volume in a sample of 57 birds.	Stewart 1962, Baldwin and Lovvorn 1994, Lovvorn and Baldwin 1996
Anas clypeata, Northern shoveler, dabbling duck	In the Netherlands (Nienhuis and Van Ierland 1978), *A. clypeata* is a rare herbivore on eelgrass.	Nienhaus and Van Ierland 1978

Anas crecca, Green-winged teal, dabbling duck	In Boundary Bay, BC, *Z. japonica* seeds (0.8%) and leaves (0.9%) made up 1.7% of esophagus dry mass in a sample of 14 birds (Baldwin and Lovvorn 1994). *Z. marina* was present in trace amounts. In the Netherlands (Nienhuis and Van Ierland 1978), *A. crecca* is a rare herbivore on eelgrass, with an estimated 25% of its diet eelgrass at Lake Grevelingen in 1978 (Nienhuis and Groenendijk 1986).	Nienhuis and Van Ierland 1978, Nienhuis and Groenendijk 1986, Baldwin and Lovvorn 1994, Lovvorn and Baldwin 1996
Anas penelope, Eurasian wigeon, dabbling duck	Johnsgard (1978) noted *Zostera* present in the diet. In the Netherlands (Nienhuis and Van Ierland 1978), *A. penelope* is sometimes a rare herbivore on eelgrass, but in other cirucumstances estimated to have a diet of 50% eelgrass (Nienhuis and Groenendijk 1986). In a study by Balsby et al. (2016) widgeon are assumed to have a diet of 100% *Zostera*. Campbell (1946) surveyed 344 gut contents and found 87 to contain fragments of *Zostera*, particularly *Z. noltei*.	Campbell 1946, Charman 1977, Johnsgard 1978, Jacobs et al 1981, Mathers and Montgomery 1996, Nienhuis and Groenendijk 1986, Hori and Hasegawa 2005
Anas platy-rhynchos, Mallard, dabbling duck	In Boundary Bay, BC, *Z. japonica* leaves, (20%), seeds (13%) and rhizomes (39%) made up the majority of esophageal contents in a sample of 20 ducks (Baldwin and Lovvorn 1994), while *Z. marina* leaves were 5%. In Chesapeake Bay (Stewart 1962), *Z. marina* composed 8 +/- 37% of gut volume in 13 birds. In Lake Akkeshi, Japan, (Hori and Hasegawa 2005) they were one of several herbivorous ducks observed eating *Z. marina* and *Z. japonica*, with highest consumption in autumn. In the Wadden Sea (Jacobs 1981), wintering birds used grubbing, digging and swimming/tearing to consume *Z. noltei*. At this site, birds removed 26% of the annual production in 1974 (50% of standing stock), and was estimated responsible for 15% of the biomass removed. In the Netherlands (Nienhuis and Van Ierland 1978 and Nienhuis and Groenendijk 1986), an estimated 1/4 of the birds' food was eelgrass, likely mostly seeds.	Stewart 1962, Baldwin and Lovvorn 1994, Lovvorn and Baldwin 1996, Jacobs et al 1981, Nienhaus and Van Ierland 1978, Nienhuis and Groenendijk 1986, Hori and Hasegawa 2005,

Anas poecilo-rhyncha, Spot-billed duck, dabbling duck	In Lake Akkeshi, Japan, *A. poecilorhyncha* were one of several herbivorous ducks observed eating *Z. marina* and *Z. japonica*, with highest consumption in autumn.	Hori and Hasegawa 2005
Anas rubripes, Black Ducks, dabbling duck	In the Chesapeake (Stewart 1962), *Z. marina* composed 26-59% (mean 42%) of gut volume in a sample of 40 birds.	Stewart 1962
Anas strepera, Gadwall, dabbling duck	In the Chesapeake (Stewart 1962), *Z. marina* composed 4-38% (mean 17%) of gut volume in a sample of 24 birds.	Stewart 1962, Johnsgard 1978
Aythya affinis, Lesser Scaup, dabbling duck	Across North American birds prior to 1938 (Cottam 1938), *Z. marina* composed 0.13% of gut contents in a sample of 1051 birds. In the Chesapeake (Stewart 1962), *Z. marina* composed 4-78% (mean 33%) of gut volume in a sample of 6 birds in salt estuary bays, and 8 +/-37% in brackish bays.	Cottam 1939, Stewart 1962
Aythya americana, Redhead, dabbling duck	Across North American birds prior to 1938 (Cottam 1938), *Z. marina* composed 0.31% of gut contents in a sample of 364 birds. In the Chesapeake (Stewart 1962), *Z. marina* composed 9-82% (mean 43%) of gut volume in a sample of 7 birds in salt estuary bays, and 8 +/- 37% in 13 birds in brackish bays.	Cottam 1939, Yocum and Keller 1961, Stewart 1962
Aythya ferina, Common Pochard, dabbling duck	In the Netherlands (Nienhuis and Van Ierland 1978), *Zostera* is estimated to compose 1/2 of birds' diets from May-August 1976 and July 1978-July 1979 (Nienhuis and Groenendijk 1986).	Nienhaus and Van Ierland 1978
Aythya marila, Greater Scaup Duck,	Across North American birds prior to 1938 (Cottam 1938), *Z. marina* composed 0.55% of gut contents in a sample of 752 birds. In the	Cottam 1939, Stewart 1962

dabbling duck	Chesapeake (Stewart 1962), *Z. marina* composed 21-74% (mean 47%) of gut volume in a sample of 15 birds in salt estuary bays, and 7-71% (mean 33%) in 9 birds in brackish bays.	
Aythya valisineria, Canvasback, dabbling duck	Across North American birds prior to 1938 (Cottam 1938), *Z. marina* composed a trace amount of gut contents in a sample of 427 birds. In the Chesapeake (Stewart 1962), *Z. marina* composed 17 +/- 65% of gut volume in a sample of 6 birds in salt estuary bays, 11 +/- 49% in 9 birds in turbid brackish bays, and 10-38% (mean 22%) in brackish bays.	Cottam 1939, Stewart 1962
Bucephala albeola, Bufflehead, diving duck	Across North American birds prior to 1938 (Cottam 1938), *Z. marina* composed a trace amount (0.11%) of gut contents in a sample of 282 birds. In the Chesapeake (Stewart 1962), *Z. marina* composed 1-35% (mean %11) of gut volume in a sample of 18 birds in brackish bays.	Cottam 1939, Stewart 1962
Bucephala clangula, Common Goldeneye, diving duck	Across North American birds prior to 1938 (Cottam 1938), *Z. marina* composed 0.71% of gut contents in a sample of 395 birds. In the Chesapeake (Stewart 1962), *Z. marina* seeds composed 14 +/- 58%) of gut volume in a sample of 7 birds in brackish bays.	Cottam 1939, Stewart 1962
Clangula hyemalis, Old Squaw/ Long tail duck, diving duck	Across North American birds prior to 1938 (Cottam 1938), *Z. marina* composed 0.60% of gut contents in a sample of 190 birds, and is noted as an important food in January and February. In the Chesapeake (Stewart 1962), *Z. marina* composed 4-78% (mean 33%) of gut volume in a sample of 6 birds in brackish bays.	Cottam 1939, Stewart 1962
Melanitta deglandi, White-winged scoter, diving duck	Across North American birds prior to 1938 (Cottam 1938), *Z. marina* composed 1.98% of gut contents in a sample of 903 birds, but 100% of the meal of one individual.	Cottam 1939

Melanitta perspi-cillata, Surf scoter, diving duck	Across North American birds prior to 1938 (Cottam 1938), *Z. marina* roots, stems and leaves composed 1.14% of gut contents in a sample of 168 birds, and was noted as likely important during migration.	Cottam 1939
Oidemia americana, American scoter, diving duck	Across North American birds prior to 1938 (Cottam 1938), *Z. marina* composed 4.03% of gut contents in a sample of 124 birds.	Cottam 1939
Oxyura jamaicensis, Ruddy Duck, diving duck	Across North American birds prior to 1938 (Cottam 1938), *Z. marina* composed 0.48% of gut contents in a sample of 163 birds.	Cottam 1939
Polysticta stelleri, Steller's eider, diving duck	Across birds sampled in Siberia and Alaska in May & June 1938 (Cottam 1938), *Z. marina* composed 1.16% of gut contents in a sample of 66 birds.	Cottam 1939
Somateria mollissima v. nigra, Pacific eider, diving duck	Across North American birds prior to 1938 (Cottam 1938), *Z. marina* composed a trace amount of gut contents in a sample of 61 birds.	Cottam 1939
Somateria mollissima dresseri, American eider, diving duck	Across birds sampled on the east coast of North America during winter prior to 1938 (Cottam 1938), *Z. marina* composed a trace amount of gut contents in a sample of 96 birds.	Cottam 1939
Somateria spectabilis, King Eider, diving duck	Across North American birds prior to 1938 (Cottam 1938), *Z. marina* composed 2.43% of gut contents in a sample of 85 birds.	Cottam 1939
Anser anser/ Anser albifrons, Greater White-	*Zostera* is noted as present in the diet of *A. anser* (Johnsgard 1978).	Johnsgard 1978

fronted goose/Greylag, goose		
Branta bernicla bernicla, Brant, goose	In the Netherlands (Nienhuis and Van Ierland 1978), *Zostera* formed 100% of diet from May -August 1976 and from July 1978-July 1979 (Nienhuis and Groenendijk 1986).	Ranwell and Downing 1959, Stewart 1962, Ladin et al 2011, Ganter 2000, Jacobs et al 1981, Charman 1977
Branta bernicla hrota, Pale-Bellied Brant, Light-Bellied Brant, goose	Birds across Europe (Robinson et al. 2004) are described qualitatively as feeding on *Zostera* regularly, but also having shifted their diet since the 1930s to diversify to eat salt marsh plants, algae, and agricultural lands. In a study by Balsby et al. (2016) geese are assumed to have a diet of 100% *Zostera*. In 1944 in North America, 9% of geese sampled for gut contents contained *Zostera* (Cottam 1938, 1944), and in England 20 of 28 stomachs (71%) contained *Zostera* fragments in 1946.	Robinson et al. 2004, Balsby et al 2016, Mathers et al 1998, Ganter 2000, Mathers and Montgomery 1996, Charman 1977, Cottam 1944
Branta bernicla nigricans, Black Brant, goose	Charman (1977) found *Zostera* to be the preferred food, but populations were not as affected by declines in *Z. marina*.	Ganter 2000
Branta canadensis, Canada Goose, goose	In Maine/New Hampshire, USA in 2002/2003, a flock of birds were observed consuming *Z. marina* in January and February and significantly damaged the bed. In Antigonish Harbor, Nova Scotia, Canada geese were regularly observed foraging on *Z. marina* beds during winter until 2001, when *Z. marina* resources declined and bird numbers fell.	Rivers and Short 2007, Seymour et al 2002
Chen canagica, Emperor Goose, goose	In Adak and Izembek Bays in Alaska, *Z. marina* made up the principal food in the stomachs of 5 birds.	Headley 1967, Johnson 1978

Fulica americana, American Coot, rail	In Yaquina Bay, coots were observed eating and foraging in *Z. marina* beds and channels (Lamberson et al. 2011). Across North America, *Z. marina* composed 0.54% of the stomach contents of 801 sampled birds, but only present in 25 sampled stomachs from November, December and January prior to 1940.	Jones 1940, Lamberson et al. 2011
Fulica atra, Eurasian Coot, rail	In the Netherlands (Nienhuis and Van Ierland 1978), estimated to compose 1/2 of diet from May-August 1976 and from July 1978-1979 (Nienhuis and Groenendijk 1986). In a study by Balsby et al. (2016) geese are assumed to have a diet of 50% Zostera.	Gayet et al 2012, Nienhaus and Van Ierland 1978, Nienhuis and Groenendijk 1986, Collinge 1936
Cygnus atratus, Black Swan, swan	In New Zealand, *Z. muelleri* is the sole food source of black swans, with 42.23% (+/- 2.3%) of biomass as shoot, 57.77 (+/- 2.3%) as rhizome. Birds consume significant amounts of both aboveground and belowground *Z. muelleri* biomass (Dos Santos 2012).	Dos Santos et al 2012, Dixon 2009
Cygnus buccinator, Trumpeter swan, swan	In Port Alberni and Comox Bay, BC, *Z. marina* fronds were observed in 33.8% of 65 collected scats from 1975-1980.	McKelvey 1981
Cygnus columbianus/Cygnus bewicki, Tundra swan/ Bewick Swan, swan	*Zostera* is noted as present in the diet of *C. columbianus* (Johnsgard 1978).	Johnsgard 1978
Cygnus cygnus, Whooper swan, swan	*Zostera* is noted as present in the diet of *C. cygnus* (Johnsgard 1978). Observed to feed on *Zostera* in Autumn in England while overwintering	Johnsgard 1978, Hori and Hasegawa 2005, Charman 1977

	(Charman 1977).	
Cygnus olor, mute swan, swan	Grazing observed in Sweden (Mathiasson et al. 1977). In the Netherlands, birds exclusively ate eelgrass from May-August 1976. In a study by Balsby et al. (2016) swans were estimated to consume hundreds of dry grams of *Zostera* daily during the fall. In England (Charman 1977), swans are noted to feed on *Zostera* in autumn while overwintering along the coasts. In the Netherlands, eelgrass was estimated to compose 100% of the mute swan’s diet (Nienhuis and Groenendijk 1986).	Mathiasson 1973, Charman 1977, Gayett et al 2012, Nienhaus and van Ierland 1978, Nienhuis and Groenendijk 1986, Balsby et al. 2016
Limosa limosa icelandica, Black-tailed Godwit, wader	One population, in one location in France, in several years, birds consumed rhizomes in place of usual invertebrates.	Robin et al. 2013
